# Lifestyle Quality Indices and Female Breast Cancer Risk: A Systematic Review and Meta-Analysis

**DOI:** 10.1016/j.advnut.2023.04.007

**Published:** 2023-04-20

**Authors:** Brianda I. Armenta-Guirado, Alejandra González-Rocha, Ángel Mérida-Ortega, Lizbeth López-Carrillo, Edgar Denova-Gutiérrez

**Affiliations:** 1 Center for Research in Nutrition and Health, National Institute of Public Health, Cuernavaca, Mexico; 2 Department of Health Sciences, University of Sonora, México; 3 Center of Population Health Research, National Institute of Public Health, Cuernavaca, Mexico

**Keywords:** Healthy lifestyle indices, BC, female, molecular subtype, menopausal status, systematic review, meta-analysis

## Abstract

Breast cancer (BC) poses an important burden of disease, which probably could be reduced by adopting healthy lifestyles like healthy body weight, healthy diet, and physical activity, among others. Many studies have reported that adherence to healthy lifestyles may decrease BC risk. The main objective of this study was to estimate a summary association of studies evaluating a healthy lifestyle index and BC risk. A systematic review and meta-analysis following the Cochrane methodology were carried out. Observational studies, including healthy lifestyle indices and their association with BC, were searched from 4 databases. For the meta-analysis, random-effects model was used to evaluate overall BC risk, BC by molecular subtype and menopausal status. Thirty-one studies were included in the systematic review, and 29 studies in the meta-analysis. When the highest vs. the lowest category to a healthy lifestyle index were compared, the study identified a 20% risk reduction for BC in prospective studies (hazard ratio [HR] 0.80 95% CI: 0.78, 0.83) and an odds ratio (OR) of 0.74 (95% CI: 0.63, 0.86) for retrospective studies. The inverse association remained statistically significant when stratified by menopausal status, except for premenopausal BC in prospective studies. Furthermore, an inverse association was found for molecular subtypes estrogen receptor (ER+)/progesterone receptor (PR+): HR = 0.68 (95%CI: 0.63, 0.73), ER+/PR-: HR = 0.78 (95% CI: 0.67, 0.90) and ER-/PR-: HR = 0.77 (95% CI: 0.64, 0.92). Most studies scored at a low risk of bias and a moderate score for the certainty of the evidence. Adherence to a healthy lifestyle reduces the risk of BC, regardless of its molecular subtypes, which should be considered a priority to generate recommendations for BC prevention at a population level.

**International prospective register of systematic reviews (PROSPERO) ID**: CRD42021267759.

**Statement of Significance**

To our knowledge, this is the first systematic review and meta-analysis to assess adherence to a healthy lifestyle index and its association with female BC, especially considering the menopausal status and molecular subtype.

## Introduction

In women, breast cancer (BC) is an important public health issue, being the most common type of cancer and the leading cause of cancer deaths worldwide [1,2]. An estimated increase of approximately 33.8% in incident cases is expected by 2040 [3]. According to global data, 645,000 premenopausal and 1.4 million postmenopausal BC cases were reported in 2018, with a higher burden of premenopausal cases observed in low- and middle-income countries than in high-income countries [4]. Although there are no worldwide incidence rates for BC molecular subtypes, some countries have information from national records. For example, in the United States, the following percentages are observed: 72.6% for luminal A (estrogen receptor +(ER+), progesterone receptor +(PR+), human epidermal growth factor receptor 2 (HER2) [ER+/PR+]), 11.2% for luminal B (estrogen receptor +, progesterone receptor -, human epidermal growth factor receptor 2 +/- [ER+/PR-]), 4.8% for HER2+, and 11.3% for triple-negative (TN) [5]. In other countries such as Mexico, there are studies that show a percentage distribution for molecular subtypes: luminal A of 43.8%, luminal B at 52.2%, HER2+ at 14.8%, and TN 22.9% in women older than 40 y [6].

Traditional behavioral risk factors such as; unhealthy body mass index (BMI), poor diets, excess alcohol, tobacco consumption, and physical inactivity have been associated with BC risk [4]. However, the combination of these factors as a lifestyle pattern may influence BC risk more than each isolated factor.

The World Cancer Research Fund/American Institute for Cancer Research (WCRF/AICR) and the American Cancer Society (ACS) have published guidelines focused on improving modifiable risk profiles like a normal range of BMI, being physically active, eating mostly plant foods, limiting the intake of red meat, alcohol, energy-dense foods while also avoiding processed meat, and soft drinks [7,8].

According to the previous information, the utilization of a score that represents a healthy lifestyle based on multiple aspects, including; a normal BMI, low alcohol intake, no tobacco use, being physically active, adhering to various aspects of a healthy diet such as the intake of fruits and vegetables, whole grains, and avoiding processed red meat (measured as a dietary pattern), would allow for the investigation of overall behavior patterns [[9], [10], [11], [12], [13], [14], [15], [16]].

Multiple studies have developed similar scores in different settings and have evaluated their association with BC [[17], [18], [19], [20], [21], [22], [23], [24], [25]], emphasizing the importance of adopting a healthy lifestyle pattern for BC prevention rather than focusing on individual factors. Although there is a review evaluating the relationship between different types of cancer, including BC, and lifestyle [26], to our knowledge, this is the first systematic review and meta-analysis to assess adherence to a healthy lifestyle index and its association with female BC at global level, especially considering the menopausal status and molecular subtype.

Thus, the main objective of our study was to systematically review and carry out a meta-analysis of the published literature reporting associations between a healthy lifestyle index and BC risk. In addition, an evaluation of the association between a healthy lifestyle index and BC by menopausal status and molecular subtype was conducted.

## Methods

This systematic review was conducted following the Cochrane Handbook for Systematic Reviews [27] and the Conducting Systematic Reviews and Meta-Analyses of Observational Studies of Etiology (COSMOS-E) [28]. The protocol was published in the International Prospective Register of Systematic Reviews (PROSPERO), ID: CRD42021267759.

### Criteria for considering studies in this review

#### Type of studies

Prospective studies (cohort, case-cohort, and nested case-control studies) and retrospective studies (population-based case-control studies and hospital-based case-control studies) published from January 2000 to February 2022 were included. Comments, letters to the editor, clinical trials, or those reports that studied BC in animals were excluded. Additionally, we did not use the statistical power that the original studies reported as an inclusion/exclusion criterion for this work.

#### Types of participants

Studies including women aged ≥20 y without a history of BC were selected for the control group. Studies reporting a histopathological diagnosis of BC or one confirmed by a self-report were included as cases.

#### Types of exposure

Prospective and retrospective studies reporting a healthy lifestyle score were included.

#### Types of outcomes

The primary outcome was the association with BC, and the secondary outcome was the association with BC by menopausal status and molecular subtypes. Studies were excluded when they did not report measurements of association [i.e., HR, OR, or RR] and 95% CI, or when studies only evaluated BC mortality, cancer recurrence, survival rates, or assessed single components of lifestyle.

### Electronic searches

A search strategy was designed with Medical Subject Headings (MesH) terms such as: “breast neoplasm,” “healthy lifestyle,” and “healthy lifestyle index.” The search was conducted in November 2021 and updated in February 2022 using 4 databases: PubMed, LILACS, CINAHL, and ScienceDirect. The detailed search strategy used per database is reported in Supplementary Table 1.

#### Reference list scanning

To exhaust our search and reduce publication bias, we examined a reference list of other reviews related to our topic in terms of healthy lifestyle indices, general cancer that include a sub-analysis on BC, and the list of the included studies.

### Selection of studies

**FIGURE 1:**
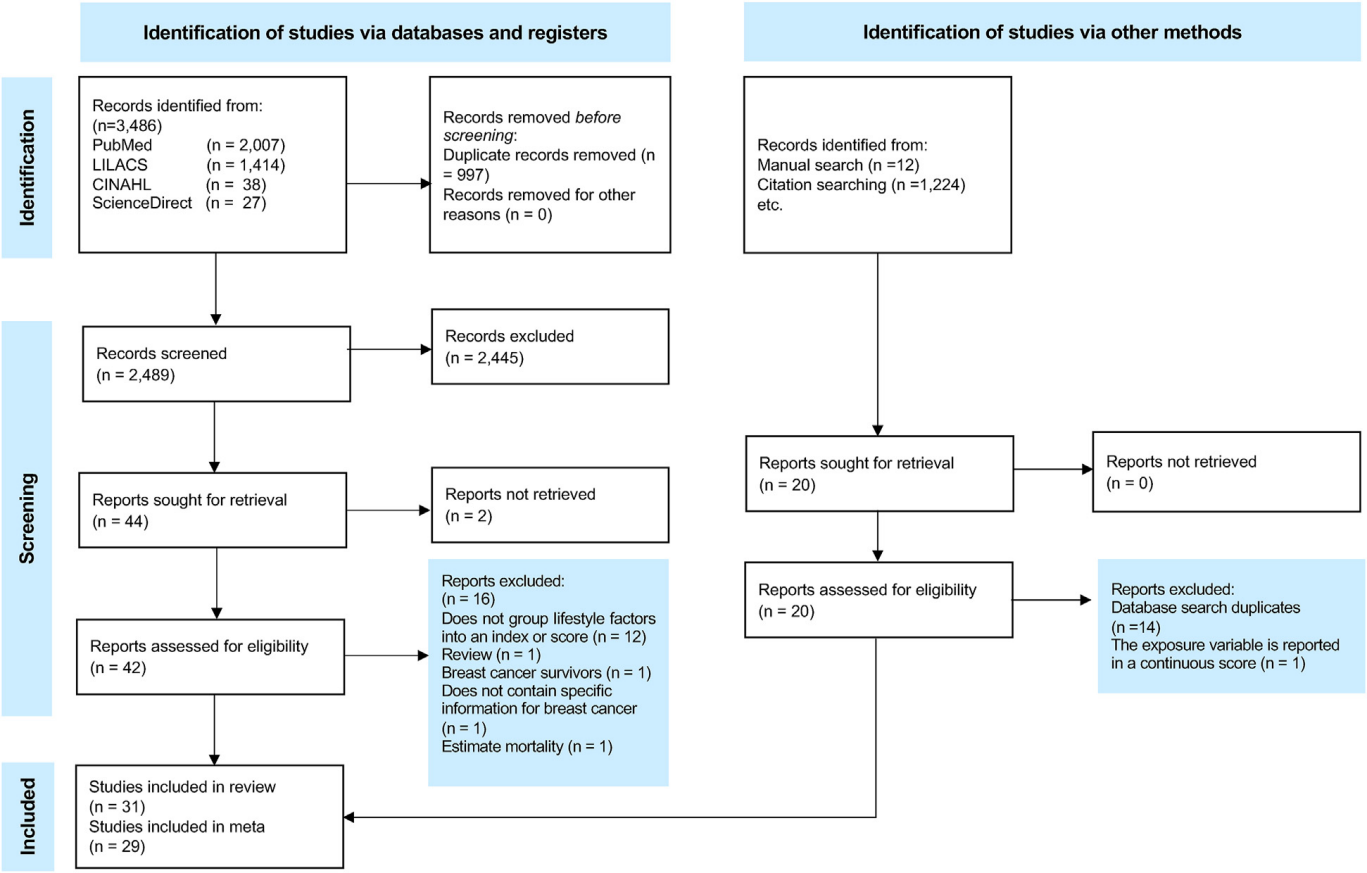
PRISMA flow chart for literature search and study selection process for inclusion in systematic review and meta-analysis of lifestyle quality indices and BC.

Two authors (BA-G and AG-R) screened titles and abstracts independently to identify relevant studies. In the first step, duplicates were removed, then titles and abstracts were screened, and finally, the full texts of the remaining studies were systematically examined to evaluate compliance with our inclusion and exclusion criteria (BA-G and AG-R). When there were disagreements, the participation of a third reviewer was required to make the final decision (ED-G). The study selection process is described in Figure 1.

### Data extraction and management

The information was extracted by 2 authors (BA-G and AG-R) based on the Population, Exposure, Comparison, and Outcome (PECO) research question. First, the characteristics of the study, such as author, publication year, and country, were extracted. Then, the *population* information was identified—sample size, age of participants, number of cases, BC type, information about molecular subtype, menopausal status, and study design. For *exposure* and *comparison*, characteristics regarding the type, number, and components of indices (diet, physical activity, tobacco consumption, alcohol consumption, BMI, nutritional supplements use, and breastfeeding) and adjustment for potential confounders. The outcomes were organized by type of design, prospective or retrospective; molecular subtype (estrogen receptor (ER)+/ progesterone receptor (PR)+, ER+/ PR-, ER-/PR-, HER2+, HER2-, and TN), and menopausal status (premenopausal and postmenopausal). The association estimates (HR, RR, or OR) and their corresponding 95% CIs were considered for the highest vs. lowest category for all comparisons between groups and were only included in multivariate-adjusted models.

### Methodological quality assessment

An adaptation of the Newcastle-Ottawa Scale (NOS) tool was used to assess the quality of the included studies [29]. This was evaluated in duplicate, independently, and then discussed by 2 authors (BA and AG-R). When there were discrepancies, 2 more experienced authors in cohort and case-control studies made the final decision (ED-G and LL-C) and supervised the judgments. For prospective studies, the domains evaluated were: 1) selection: representativeness of the exposed cohort, selection of the nonexposed cohort, ascertainment of exposure, demonstration that outcome of interest was not present at the beginning of the study; 2) comparability: comparability of cohorts on the basis of the design or analysis controlled for confounder, and 3) outcome: assessment of outcome, was follow-up long enough for outcomes to occur (10 y minimum, based on evidence from epidemiological studies), adequacy of follow-up of cohorts. The quality was classified according to the total number of stars achieved; good: 9–7 stars, fair: 6–5 stars, and poor: ≤4 stars.

For retrospective studies, the domains evaluated were: 1) selection: whether the case definition was adequate, representativeness of the cases, selection of controls, the definition of controls; 2) comparability: comparability of cases and controls based on the design or analysis; and 3) outcome: ascertainment of exposure, the same method of ascertainment for cases and controls. The quality was classified according to the total number of stars achieved: good (8-7 stars), fair (6-5 stars), and poor (≤4 stars).

The “nonresponse rate” section for retrospective studies of the original NOS tool was removed. This is because the presence of the same nonresponse rate in the comparison groups does not ensure the absence of bias but rather the nonresponse rate according to the exposure-outcome combinations [30]. The graphical summary was performed with the Review Manager 5.4 software [31].

### Meta-Analysis

#### Measurement of the association

Studies with a measurement of the association, such as OR, RR, or HR, and its confidence interval (95% CI) were included. The magnitude of the association was considered based on the comparison of the highest category to the indices (considered as a healthier lifestyle), compared with the lowest category (less healthy lifestyle). The statistical analyses were carried out using the random effects model by the restricted maximum likelihood (REML) technique. Statistical analyses were stratified as follows by epidemiological design for overall BC; by menopausal status in prospective and retrospective studies, respectively; and by molecular subtype, regardless studies design. To assess heterogeneity, the statistical inconsistency index (I^2^) was considered, ranging from 0 to 100%. To observe the magnitude of the association across the studies, forest plots with 95% CI were generated.

#### Publication bias

The risk of publication bias was assessed using funnel plots, stratifying by study design, menopausal status, and molecular subtype.

#### Sensitivity analysis

The following sensitivity analyses were performed, 1) studies that included a healthy lifestyle index based on the WCRF/AICR and/or ACS guidelines for cancer prevention, 2) studies that accounted for greater weight in the main analysis were excluded, and 3) studies that included the 5 most used variables in the indices (diet, physical activity, alcohol consumption, smoking, and BMI).

All analyses were performed with the STATA 17.0 (StataCorp. 2021. Stata Statistical Software: Release 17. College Station, TX: StataCorp LLC) software.

### Certainty of Evidence

The certainty of the evidence from the meta-analysis was performed according to the guidelines of the Grading of Recommendations, Assessment, Development, and Evaluations (GRADE) [32]. The GRADE framework classifies the quality of the evidence into 4 categories 1) high quality, further research is very unlikely to change our confidence in the magnitude of the estimated association; 2) moderate quality, further research is likely to have an important impact on our confidence in the magnitude of the estimated association and may change it; 3) low quality, further research is very likely to have an important impact on our confidence in the magnitude of the estimated association and is likely to change it; 4) very low quality, any estimate of association is very uncertain [33]. This evaluation was conducted using the software GRADE Pro Version 3.6 [34].

## Results

A total of 3,486 published studies were identified from databases, and 42 publications met the inclusion criteria. Sixteen publications were excluded because lifestyle factors were not grouped using an index or score (n = 12). Additionally, 5 studies were retrieved from the reference list scanning and met the inclusion criteria. Therefore, 31 publications were finally included in the systematic review (Figure 1**)**, of which 24 were prospective studies: 21 cohort studies [10,11,13,17,[19], [20], [21], [22],[35], [36], [37], [38], [39], [40], [41], [42], [43], [44], [45], [46], [47]], one case-cohort study [48], and 2 nested case-control studies [12,49]. Seven studies were retrospective with a case-control design:6 population-based case-control studies [[14], [15], [16],^18^,^23^,^25^] and one hospital-based case-control study [24] (Table).

**TABLE:** Characteristics of prospective and retrospective studies included in a systematic review of lifestyle quality indices and BC

Author/Year/ Country	Sample size/Age	N of cases	Type of indices or scores	Components	HR/OR Overall BC (Highest vs. Lowest category)	HR/OR Molecular subtype (Highest vs. Lowest category)	HR/OR Menopausal status (Highest vs. Lowest category)	Adjusted variables	Risk of bias summary NOS tool
***Prospective studies***									
Arthur, 2018 USA	131,833 Cases: 63 (57,69) y Noncases: 63 (57,68) y	8,168	Healthy lifestyle index (HLI)	1. Diet: a priori 2. Physical activity 3. Alcohol consumption 4. Smoking 5. BMI	NA	**ER+/PR+** HR = 0.63 (95% CI: 0.57, 0.69) **ER+/PR-** HR = 0.92 (95% CI: 0.74, 1.14) **ER-/PR-** HR = 0.86 (95% CI: 0.69, 1.09) **HER2+** HR = 0.70 (95% CI: 0.55, 0.90) **HER2-** HR = 0.67 (95% CI: 0.06, 0.73) **TN** HR = 0.78 (95% CI: 0.58, 1.07)	**Postmenopausal:** HR = 0.70 (95% CI: 0.64, 0.76)	Age at entry, ethnicity, height, education, family history of BC in first-degree relative, age at menarche, parity, breastfeeding, history of mammograms, age at menopause, hormone replacement therapy use, oral contraceptive use, history of benign breast disease, nonalcohol energy intake; for the stratified analyses, the models included all these variables except the stratification variable.	Good ★★★★★★★★
Arthur, 2018 Canada	131,833 67 (59-75) y	410	Healthy lifestyle index (HLI) score	1. Diet: *a priori* 2. Physical activity 3. Alcohol consumption 4. Smoking 5. BMI	NA	NA	**Postmenopausal:** HR = 0.70 (95% CI: 0.53, 0.93)	Education, nonalcohol energy intake, age at menarche, parity, breastfeeding, menopausal status, HRT use ever, oral contraceptive use, family history of BC in a first-degree relative. When the individual components were included as the main exposures, the models were also adjusted for diet, alcohol, physical activity, BMI, smoking, unless included as the main exposure.	Good ★★★★★★★★
Arthur, 2020 England	146,326 Premenopausal: Cases: 46 (43,48) y Noncases: 45 (43-49) y Postmenopausal Cases: 61 (58,64) y Noncases: 61 (56,64) y	3,422	Healthy Lifestyle Index (HLI) based on WCRF/AICR 2018	1. Diet: *a priori* 2. Physical activity 3. Alcohol consumption 4. Smoking 5. BMI: postmenopausal women only 6. WC: postmenopausal women only	NA	NA	**Premenopausal:** HR = 0.78 (95% CI: 0.64, 0.94) **Postmenopausal:** HR = 0.69 (95% CI: 0.63, 0.77)	Age at recruitment, socioeconomic status, age at menarche, parity, age at first live birth, ever use of hormone replacement therapy (postmenopausal women only), ever use of oral contraceptives, history of mammograms, age at menopause (postmenopausal women only), family history of BC, BMI (premenopausal women only), the first 5 genetic principal components genotyping batch, as well as PRS and HLI, unless (for the latter 2 variables).	Good ★★★★★★★★
Barrios-Rodríguez, 2020 Spain	10,930 0,7 points: 35.0 ± 10.6 y ≤3 points: 32.0 ±8.9 y 3, ≤5 points: 34.6 ±10.3 y >5 points: 39.9 ±11.3 y	119	WCRF/AICR 2018	1. Diet: *a priori* 2. Physical activity 3. Alcohol consumption 4. BMI 5. Breastfeeding	HR = 0.62 (95% CI: 0.27, 1.43)	NA	**Premenopausal:** HR = 0.67 (95% CI: 0.30,1.47) **Postmenopausal** HR = 0.27 (95% CI: 0.08,0.93)	Total energy intake, years at university, smoking status, family history of BC, menopause, age at menarche, age at first pregnancy, use of hormone replacement therapy, and oral contraceptive use. Analysis for overall BC was additionally adjusted for age at menopause. For postmenopausal women, in addition to age of menopause, also adjusted for time since recruitment.	Good ★★★★★★★★★
Catsburg, 2014 Canada	49,613 Cases: 50.0 (45.3,54.9) y Noncases: 48.8 (44.1,53.7) y	2,503	ACS WCRF/AICR 2007	ACS: 1. Diet: *a priori* 2. Physical activity 3. Alcohol consumption 4. BMI WCRF: 1. Diet: *a priori* 2. Physical activity 3. BMI	ACS: HR = 0.69 (95% CI 0.49, 0.97) WCRF/AICRF: HR = 0.79 (95% CI: 0.57, 1.10)	NA	NA	Age, age at menarche, use of oral contraceptives, use of hormone therapy, age at first live birth, family history of BC, history of breast disease, menopausal status at baseline, and study center.	Good ★★★★★★★★★
Chen, 2021 Norway	96,869 51.6 ±6.4 y	3,397	Healthy Lifestyle Index (HLI) based on WCRF/AICR 2018 and scientific knowledge	1. Diet: *a priori* 2. Physical activity 3. Alcohol consumption 4. Smoking 5. BMI	NA	NA	**Postmenopausal:** HR = 0.83 (95% CI: 0.76, 0.91)	Education, height, age at menarche, use of oral contraceptives, parity, breastfeeding, use of hormone replacement therapy, family history of BC in a first-degree relative.	Good ★★★★★★★★★
Cifu, 2018 USA	106,126 Q1: 62.1 ±5.3 y Q5: 62.3 ±5.4 y	7,088	American Cancer Society (ACS) guidelines	1. Diet: *a priori* 2. Physical activity 3. Alcohol consumption 4. Smoking 5. BMI	HR = 0.76 (95% CI: 0.70, 0.82)	NA	NA	Medical history and treatment: first-degree relative with BC, ever/never use of menopausal hormone therapy, general health status, education level.	Good ★★★★★★★★★
Dartois, 2014 French	64,732 43 to 68 y	3,483	Health index French National Program for Health and Nutrition/WHO.	1. Diet: *a priori* 2. Physical activity 3. Alcohol consumption 4. Smoking 5. BMI	HR = 0.81 (95% CI: 0.73, 0.89)	NA	**Premenopausal** HR = 0.80 (95% CI: 0.58, 1.12) **Postmenopausal** HR = 0.87 (95% CI: 0.74, 1.03)	Level of education, residence, first-degree family history of any cancer, professional activity, use of oral contraceptives, menopausal status, use of menopausal hormone therapy, age at menarche, number of children, age at first full-term pregnancy, more than one child with the first before age 30. Models for the individual effect of each of the 5 lifestyle characteristics scores were further adjusted for the 4 other characteristics.	Fair ★★★★★★
Guinter, 2018 USA	39,104 ≤ 2 (high estrogenic potential): 61.9 (61.7, 62.0)^3^ y ≥ 5 (low estrogenic potential): 63.0 (62.9, 63.2)^3^ y	1,576	Estrogen-related lifestyle score (ERLS)	1. Estrogenic Diet: *a posteriori* 2. Physical activity 3. Alcohol consumption 4. BMI	NA	**ER+** HR = 0.63 (95% CI: 0.51, 0.77) **ER-** HR = 0.84 (95% CI: 0.52, 1.37)	**Postmenopausal:** HR = 0.77 (95% CI: 0.67, 0.89)	Demographic factors of age, race/ ethnicity, study center were included in the multivariable-adjusted models, along with total energy intake. Further adjustment for PMH use, family history of BC, education, BMI at age 20, bilateral oophorectomy, parity, and age at menopause.	Good ★★★★★★★★
Harris, 2016 Sweden	31,514 0-2: 61.2 ± 9.2 y 3: 61.4 ± 8.9 y 4: 61.4 ± 8.8 y 5: 61.6 ± 9.0 y 6-7: 62.0 ± 9.3 y	1,388	WCRF/AICR 2007	1. Diet: *a priori* 2. Physical activity 3. Alcohol consumption 4. BMI 5. Dietary supplements	HR = 0.49 (95% CI: 0.35, 0.70)	**ER+/PR+** HR = 0.44 (95% CI: 0.27, 0.70) **ER-/PR-** HR = 0.90 (95% CI: 0.33, 2.42)	NA	Age, height, education, oral contraceptive use, hormone replacement therapy use, age at menarche, menopausal status/age at menopause, family history of BC, history of benign breast disease, and smoking status.	Good ★★★★★★★★★
Hastert, 2013 USA	30,797 50-76 y	899	WCRF/AICR 2007	1. Diet: *a priori* 2. Physical activity 3. Alcohol consumption 4. BMI	NA	NA	**Postmenopausal:** HR = 0.40 (95% CI: 0.25, 0.65)	Education, race, age at menarche, age at birth of the first child, years of combined estrogen plus progestin hormone therapy use, age at menopause, receipt of a mammogram in the 2 y before baseline, history of BC in a first-degree relative using the categories, as well as adjustment for kilocalories of average daily energy intake.	Good ★★★★★★★★
Kabat, 2015 USA	189,575 0,3: 61.1 ±5.4 y 4-5: 61.7 ±5.4 y 6: 62.1 ±5.4 y 7: 61.4 ±5.3 y 8-11: 62.7 ± 5.3 y	9,072	American Cancer Society (ACS) 2006	1. Diet: *a priori* 2. Physical activity 3. Alcohol consumption 4. BMI	HR = 0.81 (95% CI: 0.76, 0.87)	NA	NA	Age, educational level, ethnicity, smoking status, marital status, and energy intake. Breast, ovarian, and endometrial cancers also were adjusted for menopausal status, age at menarche, age at first birth, parity, and hormone therapy use. BC also was adjusted for family history of BC in a first-degree relative and mammographic screening	Good ★★★★★★★★
Karavasiloglou, 2019 European countries	261,428 51.7 ±9.9 y	1,277	WCRF/AICR 2018	1. Diet: a priori 2. Physical activity 3. Alcohol consumption 4. BMI 5. Breastfeeding	HR = 0.98 (95% CI: 0.80, 1.22)	NA	**Premenopausal** HR = 1.04 (95% CI: 0.95, 1.15) **Postmenopausal** HR = 0.94 (0.87, 1.02)	Highest level of attained education, smoking status, total dietary energy consumption, a priori determined confounders including the presence of chronic diseases at recruitment, age at menarche, age at first full-term pregnancy, menopausal status, ever use of oral contraceptive pills, ever use of menopausal hormone therapy.	Good ★★★★★★★★★
Lavalette, 2018 French	40,542 54.6 ±8.7	488	WCRF/AICR 2018	1. Diet: a priori 2. Physical activity 3. Alcohol consumption 4. BMI 5. Breastfeeding	HR = 0.64 (95% CI: 0.46, 0.89)	NA	NA	Multivariable models were adjusted for age, height, smoking status, number of dietary records, energy intake without alcohol, family history of cancer among first-degree relatives, higher education, body mass index, physical activity, the number of biological children, menopausal status at baseline, hormonal treatment for menopause, oral contraception. Adjustments for BMI and physical activity were not performed for scores in which they were included as components.	Good ★★★★★★★★
1Lofterød, 2020 Norway	17,145 41.7 ±13.8 y	574	WCRF/AICRF 2018	1. Physical activity 2. Alcohol consumption 3. Smoking 4. BMI 5. Hypertension favorable	HR = 1.34 (95% CI: 0.97,1.85)	NA	**Premenopausal** HR = 0.83 (0.53, 1.31) **Postmenopausal** 2.13 (1.23, 3.69)	Age, age at menarche, and the number of live births. Stratified by MHT users and non-users	Good ★★★★★★★
McKenzie, 2015 European countries	242,918 ≤5 points: 53 (50,58)^4^ y (6,10 points): 53 (50,59)^4^ y (11,15 points): 54 (50,60)^4^ y ≥16 points 53 (50,60)^4^ y	7,756	Healthy lifestyle index score (HLIS)	1. Diet 2. Physical activity 3. Alcohol consumption 4. Smoking 5. BMI	NA	**ER+/PR+** HR = 0.81 (95% CI: 0.67, 0.98) **ER-/PR-** HR = 0.06 (95% CI: 0.40, 0.90)	**Postmenopausal** HR = 0.74 (95% CI: 0.66, 0.83)	Height, age at menarche, age at full-term pregnancy, education, oral contraceptive use, hormone replacement therapy use, breastfeeding, total energy intake excluding alcohol.	Good ★★★★★★★★★
Nomura, 2016 USA	36,626 IWHS cohort: 61.7± 6 4.2 y BC cases: 61.6 ±6 4.1 y	3,189	WCRF/AICRF 2007	1. Diet: a priori 2. Physical activity 3. Alcohol consumption 4. BMI	NA	NA	**Postmenopausal** HR = 0.76 (95% CI: 0.67, 0.87)	Age, smoking status, education, hormone replacement therapy usage. Additional covariates included family history of BC, menarche age, menopause age, and parity, except in models where associations were evaluated according to that non-modifiable risk factor. Similarly, BMI/alcohol/physical activity variables were included in models where BMI/alcohol/physical activity score were not the exposure of interest.	Good ★★★★★★★★
Nomura, 2016 USA	49,103 <3 score: 38.4 ± 10.0 y 3-4 score: 38.3 ± 10.7 y >4 score: 36.9 ± 10.6	1,567	WCRF/AICRF 2007	1. Diet: a priori 2. Physical activity 3. Alcohol consumption 4. BMI 5. Dietary supplements	HR = 0.84 (95% CI: 0.65, 1.08)	**ER+/PR+** HR = 0.97 (95% CI: 0.67, 1.42) **ER+ or PR-** HR = 1.33 (95% CI: 0.70, 2.53) **ER-/PR-** HR = 0.32 (95% CI: 0.14, 0.74)	NA	All adjusted models included age, geographic region of residence, daily caloric intake, smoking, family history of BC, education, menopausal status, duration of postmenopausal female hormone supplement use, duration of oral contraceptive use, and parity. When diet score and individual recommendations were evaluated, BMI, alcohol intake, physical activity level, and sedentary time were included in models where the variable was not part of the score being evaluated (diet score example: BMI, physical activity, and sedentary time were included, but alcohol was not because it was included in the score.	Good ★★★★★★★★★
Peila, 2021 European countries	Premenopausal: Cases: 46.5 ±4.0 y Noncases: 46.2 ±4.0 y Postmenopausal: Cases: 60.5 ±5.1 y Noncases: 59.7 ±5.5 y	1,796	Healthy lifestyle index (based on WCRF-2018)	1. Diet: *a priori* 2. Physical activity 3. Alcohol consumption 4. Smoking 5. BMI 6. Waist circumference	NA	NA	**Premenopausal** HR = 0.87 (95% CI: 0.67, 1.12) **Postmenopausal** HR = 0.76 (95% CI: 0.64, 0.91)	Age at enrollment, socioeconomic status, race, height, family history of BC, use of hormone replacement therapy, use of oral contraceptive, number of live births, history of mammogram screening, and age at menopause.	Good ★★★★★★★★
Rasmussen-Torvik, 2013 USA	7,223 53.7 ±5.7 y	526	AHA 2020 Strategic Impact Goals	1. Diet: *a priori* 2. Physical activity 3. Smoking 4. BMI 5. Total cholesterol 6. Blood pressure 7. Fasting plasma glucose	HR = 0.52 (95% CI: 0.26, 1.03)	NA	NA	Age, race, and ARIC center	Good ★★★★★★★
Romaguera, 2012 European countries	260,098 53.0 ±9.8 y	9,358	WCRF/AICR 2007	1. Diet: *a priori* 2. Physical activity 3. Alcohol consumption 4. BMI 5. Breastfeeding	HR = 0.84 (95% CI: 0.78, 0.90)	NA	NA	Educational level, presence of chronic diseases at baseline, smoking status and intensity of smoking, menopausal status, ever use of hormone replacement therapy, ever use of contraception pills, age at menarche, parity, age at first full-time pregnancy, and total energy.	Good ★★★★★★★★★
Thomson, 2014 USA	65,838 0-3 score: 62.8 ±7.2 y 4-5 score: 63.5 ±7.3 6-8: 63.4 ±7.4	3,549	American Cancer Society (ACS) 2006-2012	1. Diet: *a priori* 2. Physical activity 3. Alcohol consumption 4. BMI	NA	NA	**Postmenopausal** HR = : 0.78 (95% CI: 0.67, 0.92)	Age, education, smoking pack-years, nonsteroidal anti-inflammatory drug use, aspirin use, unopposed estrogen use, estrogen + progestin use, multivitamin use, race/ethnicity, total energy intake, parous, mammogram, colonoscopy or sigmoidoscopy, family history of cancer, and having a current healthcare provider.	Good ★★★★★★★★
Warren, 2016 USA	24,613 Cohort: 50 (11) y	352	American Cancer Society (ACS) 2012	1. Diet: *a priori* 2. Physical activity 3. Alcohol consumption 4. Smoking 5. BMI	HR = 1.28 (95% CI: 0.52, 3.19)	NA	NA	Race, enrollment source, family history of cancer, insurance coverage, education, income, marital status, neighborhood deprivation index, smoking status, total energy intake, postmenopausal hormone use, and for menopausal status.	Good ★★★★★★★
Xu, 2018 Canada	157,87 C1: 51.3 ±9.1 y C2: 50.8 ±9.3 y C3: 49.4 ±9.1 y	454	WCRF/AICR 2007	1. Diet: *a priori* 2. Physical activity 3. Alcohol consumption 4. BMI 5. Dietary supplements	HR = 0.86 (95% CI: 0.68, 1.09)	NA	NA	Age, sex, marital status, education level, employment status, annual household income, tobacco exposure, first-degree family history of cancer, and personal history of chronic disease, as well as hormone replacement therapy	Good ★★★★★★★★
***Retrospective studies***									
2Castelló, 2015 Spain	1,946 22-71 y	973	WCRF/AICR 2007	1. Diet: *a priori* 2. Physical activity 3. Alcohol consumption 4. BMI 5. Breastfeeding	OR = 0.34 (95% CI:0.18, 0.63)	**HR+ or ER+/PR+** OR = 0.28 (95% CI: 0.14, 0.54) **HER+** OR = 0.24 (95% CI: 0.09, 0.60) **TN** OR = 0.43 (95% CI: 0.22, 0.83)	**Premenopausal** OR = 0.38 (95 % CI: 0.17, 0.81) **Postmenopausal** OR = 0.28 (95 % CI: 0.10, 0.81)	All models included the following potential confounders: total calorie intake, smoking habit, age at first delivery, education, history of breast problems, family history of BC, and menopausal status. Models for noncompliance with individual recommendations were also adjusted for the overall score obtained by adding up all the individual recommendations except the one under study. Multinomial logistic regression models were used to evaluate the association of the WCRF/ AICR score/individual recommendations with each of the aforementioned intrinsic BC subtypes. These models were adjusted for age, hospital, and the same set of potential confounders described above.	Good ★★★★★★★
Fanidi, 2015 Mexico	2,074 Cases: 52 (39.1-65.8)^5^ y Controls: 51 (39.2-65.3)^5^ y	1,000	WCRF/AICR 2007	1. Diet: *a priori* 2. Physical activity 3. Alcohol consumption 4. BMI 5. Breastfeeding	OR = 1.04 (95% CI: 0.78, 1.41)	NA	**Premenopausal** OR = 1.17 (95 % CI: 0.75,1.82) **Postmenopausal** OR = 0.97 (95 % CI: 0.64,1.46)	Matching accounted for age category, healthcare system, and region (model 1). Confounding factors were then included in the model (model 2), i.e., family history of BC, age at menarche, age at first pregnancy, parity, socioeconomic status, hormone replacement therapy, and total energy consumption. Smoking status and use of oral contraceptives were not included in the different models because their inclusion in the statistical model did not change the results.	Good ★★★★★★★★
Ghosn, 2020 Iranian	1,050 Cases: 65 ± 11 y Controls: 61 ± 10 y	350	Healthy lifestyle score (HLS)	1. Diet: *a priori: HEI-2010* 2. Physical activity 3. Smoking	OR = 0.62 (95% CI: 0.04,0.94)	NA	**Premenopausal** OR = 1.59 (95% CI: 0.45, 5.59) **Postmenopausal** OR = 0.56 (95% CI: 0.36,0.88)	Age, residence, marital status, SES, family history of BC, menopausal status, breastfeeding, history of the disease, and supplement use were adjusted in the first model. BMI was additionally adjusted in the second model.	Good ★★★★★★★★
Khalis, 2019 Morocco	600 Cases: 49.7 ± 11.3 y Controls: 49.5 ± 11.5 y	300	Healthy Lifestyle Index (HLI)	1. Diet: *a priori* 2. Physical activity 3. Alcohol consumption 4. Smoking 5. BMI 6. Breastfeeding	OR = 0.15 (95% CI: 0.07,0.32)	NA	**Premenopausal** OR = 0.22 (95% CI: 0.10, 0.49) **Postmenopausal** OR = 0.11 (95% CI: 0.04,0.30)	Age, number of live births, menopausal status combined with age at menopause, and postmenopausal, history of oral contraceptives, family history of BC, wealth score, age at first full-term pregnancy, and energy intake, when appropriate.	Good ★★★★★★★
McKenzie, 2014 New Zealand	1,123 Premenopausal Māori: Cases: 43.3 ± 7.1 y Controls: 42.4 ± 6.4 y Premenopausal Non-Māori: Cases: 44.6 ± 5.4 y Controls: 44.9 ± 5.7 y Postmenopausal Māori: Cases: 59.5 ± 8.3y Controls: 58.6 ± 7.6 Postmenopausal Non-Māori: Cases: 64.6 ± 10.0y Controls: 63.5 ± 9.1y	1,093	Healthy lifestyle index score (HLIS)	1. Diet: *a priori* 2. Physical activity 3. Alcohol consumption 4. Smoking 5. BMI 6. Breastfeeding	NA	NA	**Premenopausal** OR = 1.23 (95% CI: 0.83,1.83) **Postmenopausal** OR = 0.86 (95% CI: 0.67,1.11)	Age, parity, age at menarche, history of maternal BC, oral contraceptive use, HRT use, diabetes, and socioeconomic position (SEP).	Good ★★★★★★★★
Romaguera, 2017 Spain	4,774 20-85 y	1,343	WCRF/AICRF 2007	1. Diet: *a priori* 2. Physical activity 3. Alcohol consumption 4. BMI	OR = 0.76 (95% CI: 0.63,0.92)	**HR+** OR = 0.84 (95% CI: 0.68,1.03) **HER2+** OR = 0.57 (95% CI: 0.39,1.82) **TN** OR = 0.93 (95% CI: 0.54,1.59)	**Premenopausal** OR = 0.97 (95 % CI: 0.68,1.40) **Postmenopausal** OR = 0.64 (95 % CI: 0.51,0.81)	Age, educational level, area, family history of each cancer, smoking status and total energy intake, menopausal status, oral contraceptive use, hormone replacement therapy use, age at menarche, age at first pregnancy, and the number of children. Models 1 and 2 were also run after stratification according to a series of key variables that might influence the association between the WCRF/AICR score and cancer, including tumor subtype, smoking status, and menopausal status.	Good ★★★★★★★★
Sánchez-Zamorano, 2011 México	2,074 Cases: 52 ± 10 y Controls: 51 ±9 y	1,000	Healthy Lifestyle Index	1. Dietary pattern: *a posteriori* 2. Physical activity 3. Alcohol consumption 4. Smoking	NA	NA	**Premenopausal** OR = 0.50 (95% CI: 0.29,0.84) **Postmenopausal** OR = 0.20 (95% CI: 0.11,0.37)	Models accounted for matching by age category, health care system, region, and factors adjusted for in previous literature such as: socioeconomic status, breastfeeding, age at menarche, age at menopause, BMI, family history of BC in first-degree relatives, personal history of diabetes, waist-to-hip ratio, height, daily intake of folate, and total calories.	Good ★★★★★★★★

N: number; HR: hazard ratio; OR: odds ratio; BMI: Body Mass Index; USA: United States of America; NA: not available; 95% CI: 95% confidence interval; y: years; WC: waist circumference; WCRF: World Cancer Research Fund; AICR: American Institute for Cancer Research; ER: estrogen receptor; PR: progesterone receptor; HER2: human epidermal growth factor receptor 2; TN: triple-negative; ACS: American Cancer Society; WHO: World Health Organization; AHA: American Heart Association; ACS: American Cancer Society; WHO: World Health Organization; AHA: American Heart Association; IWHS: Iowa Women's Health Study: ARIC: Atherosclerosis Risk In Communities; PRS: polygenic risk score; PMH: Postmenopausal hormone; HRT: Hormone replacement therapy; SES: socioeconomic status; BC: BC; SEP: socioeconomic position; HEI: Healthy Eating Index; Q1: quintile 1; Q3: quintile 5.

Age is shown as mean ±standard deviation, or as median and (interquartile range); or as a hyphen-separated age range.

1 The authors evaluated the association considering the category of higher adherence to the indices as a healthier lifestyle, compared to the category of lower adherence.

2 For the meta-analysis, the inverse point estimate was calculated for this study since the authors considered the category with the highest adherence to a healthy lifestyle index as the reference category.

3 Age shown as mean and 95% confidence interval.

4 Age shown as medians and (25th,75th percentiles).

5 Age shown as medians and (10th,90th percentiles).

### Healthy lifestyle index characteristics

Fourteen prospective studies were based on the WCRF/AICR guidelines, either in its 2007 or 2018 version. Five prospective studies follow the ACS-2006 or 2012 guidelines [12,17,20,41,43], and the remaining studies considered other healthy lifestyle recommendation guidelines or were not based on specific guidelines but instead contemplated local evidence-based recommendations for cancer prevention [19,42,46,[48], [49], [50]]. For retrospective studies, 3 of them were based on the WCRF/AICR-2007 guidelines [[14], [15], [16]], and the remaining 4 considered a combination of different evidence-based cancer prevention guidelines [18, [23], [24], [25]]. The main components included in the indices were: diet, physical activity, alcohol consumption, and BMI, followed by tobacco consumption, supplements use, and breastfeeding. Most of the prospective studies included 5 variables in the index [17,19,43,44,46,48,50]: diet, physical activity, alcohol and tobacco consumption, and BMI, whereas most of the retrospective studies included between 5 [15,16] and 6 [18,51] variables in the index: diet, physical activity, alcohol consumption, BMI, breastfeeding and/or supplement use (Table).

#### Dietary patterns

All healthy lifestyle indices included diet as a variable, except for the study of Lofterød et al. 2020 [36]. For diet quality assessment, 28 studies [10,11,[20], [21], [22], [23],^35^,[38], [39], [40], [41], [42],^12^,[43], [44], [45], [46], [47], [48],^50^,^51^,[13], [14], [15], [16], [17], [18], [19]] used an *"a priori"* approach, whereas only 2 studies used *"a posteriori"* methodology [25,49]. In general, the dietary patterns within the lifestyle indices were composed as follows: eat a diet rich in whole grains, fruits, vegetables, legumes, low-fat dairy products, and polyunsaturated fats; reduce the consumption of fast food, salt, and salt-preserved foods, red and processed meats, limit the consumption of energy-dense foods, saturated fats and avoid sugars and sugary drinks.

### Healthy lifestyle indices and BC

Seven prospective studies showed an inverse statistically significant association between a greater adherence to healthy lifestyle indices and BC overall [12,13,17,19,20,35,40], data oscillated from an HR of 0.49 (95% CI: 0.35,0.70) to an HR of 0.84 (95% CI: 0.78,0.90). Five retrospective studies provided information on the overall association of lifestyle indices and BC [[14], [15], [16],^23^,^24^]; 4 of these studies observed a statistically significant inverse association, with an OR ranging from 0.15 (95% CI: 0.07, 0.32) to 0.76 (95% CI: 0.63, 0.92) (Table).

#### Healthy lifestyle indices and BC by menopausal status

Fourteen prospective studies provided information regarding menopausal status [10,11,19,21,22,38,41,44,[46], [47], [48], [49], [50]]. Only one of these [10] found a statistically significant inverse association between a healthy lifestyle index and BC in premenopausal women (HR = 0.78, 95% CI: 0.64, 0.94), whereas 11 studies [10,11,22,38,41,44,[46], [47], [48], [49], [50]] found an inverse and significant association for postmenopausal BC risk and a healthy lifestyle index. On the other hand, 7 retrospective studies presented information about the menopausal status [[14], [15], [16],^18^,[23], [24], [25]], 3 of them found a statistically significant negative association among premenopausal BC [15,24,25] with ORs ranging from 0.22 (95% CI: 0.10, 0.49) to 0.50 (95% CI: 0.29, 0.84) (Table).

#### Healthy lifestyle indices and BC by molecular subtype

Five prospective studies included information about the following molecular subtypes: ER+/ PR+, n = 4; ER+/ PR-, n = 3; ER-/PR-, n = 5; HER2+: n = 1; HER2-: 1 and TN: n = 1 [35,39,46,49,50]. Two retrospective studies contained information regarding BC molecular subtypes ER+/PR+, HER2 +, and TN [14,15]. Both the prospective and retrospective studies that provided information on healthy lifestyle indices and HER2+ BC molecular subtype showed a statistically significant negative association (HR = 0.70; 95% CI: 0.55, 0.90 and OR = 0.24; 95% CI: 0.09, 0.60, respectively [14,46]), except for one (14). No association was found in the single prospective study that included TN molecular subtype [46], whereas one of the 2 retrospective studies showed a negative association between TN BC molecular subtype and healthy lifestyle indices (OR = 0.43; 95% CI: 0.22, 0.83) with statistical significance [15] (Table).

### Methodological quality assessment

**FIGURE 2:**
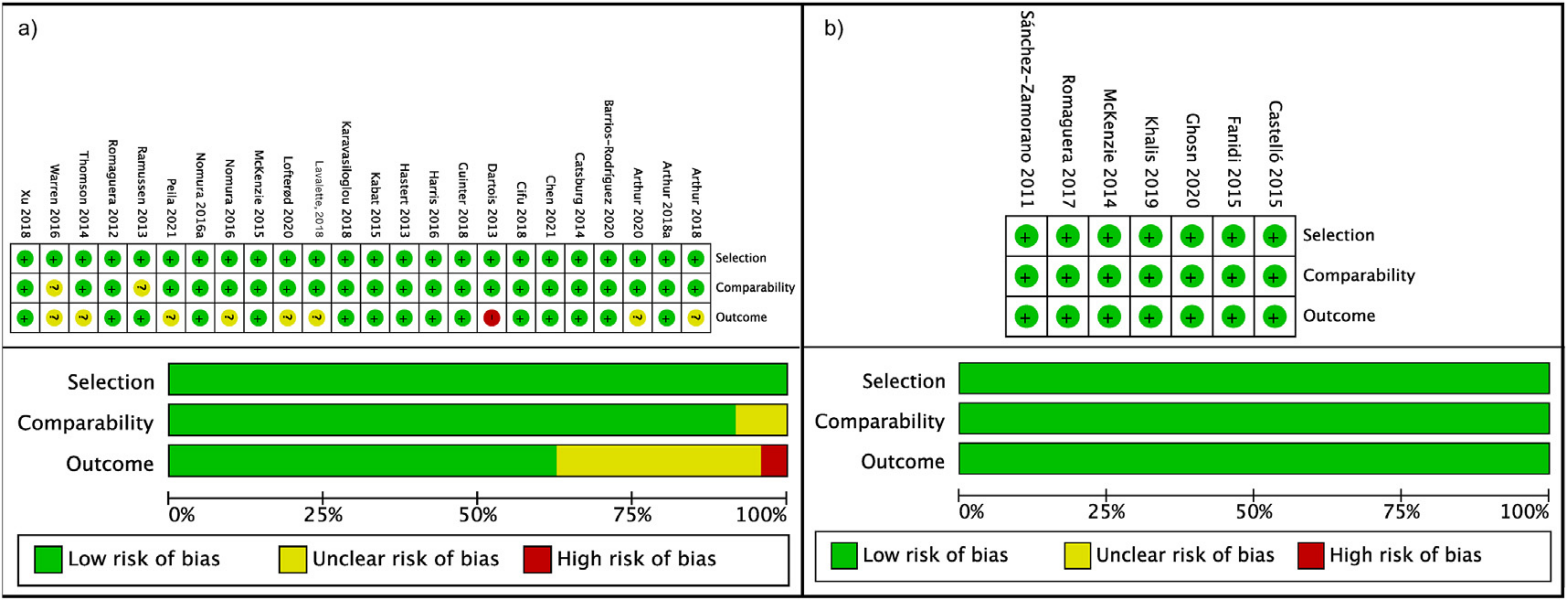
Summary graph of risk of bias from prospective and retrospective studies. a) Summary graph of risk of bias from prospective studies. b) Summary graph of risk of bias from retrospective studies.

The quality assessment summary and graph are presented in Figure 2. Our analysis identifies that none of the prospective studies had poor quality of evidence (Supplemental Table 2). The range of the quality was between 9 stars (good quality) for 7 studies [11,17,21,35,39,40,50] and 6 stars (fair quality) for 1 study [19]. A total of 16.7% of the studies were downgraded due to the method used to ascertain exposure since the instruments were not validated for the study population [20,36,48,49]. Regarding the outcome domain, 12.5% of the studies had a fair quality because they obtained the information by self-report or the information was unclear [19,39,41]. Seven of the 24 prospective studies did not have a long enough follow-up for BC cases to occur [10,13,36,47,48].

Regarding retrospective studies, all the studies presented good quality. Five studies achieved 8 stars [14,16,18,23,25] and 2 studies [15,24] achieved 7 stars. The principal concern with these studies was the selection of the control domain, which was hospital or clinical based-controls [15,24], whereas 71.4% of the studies reported population or community controls, Supplemental Table 2.

### Meta-analysis

**FIGURE 3:**
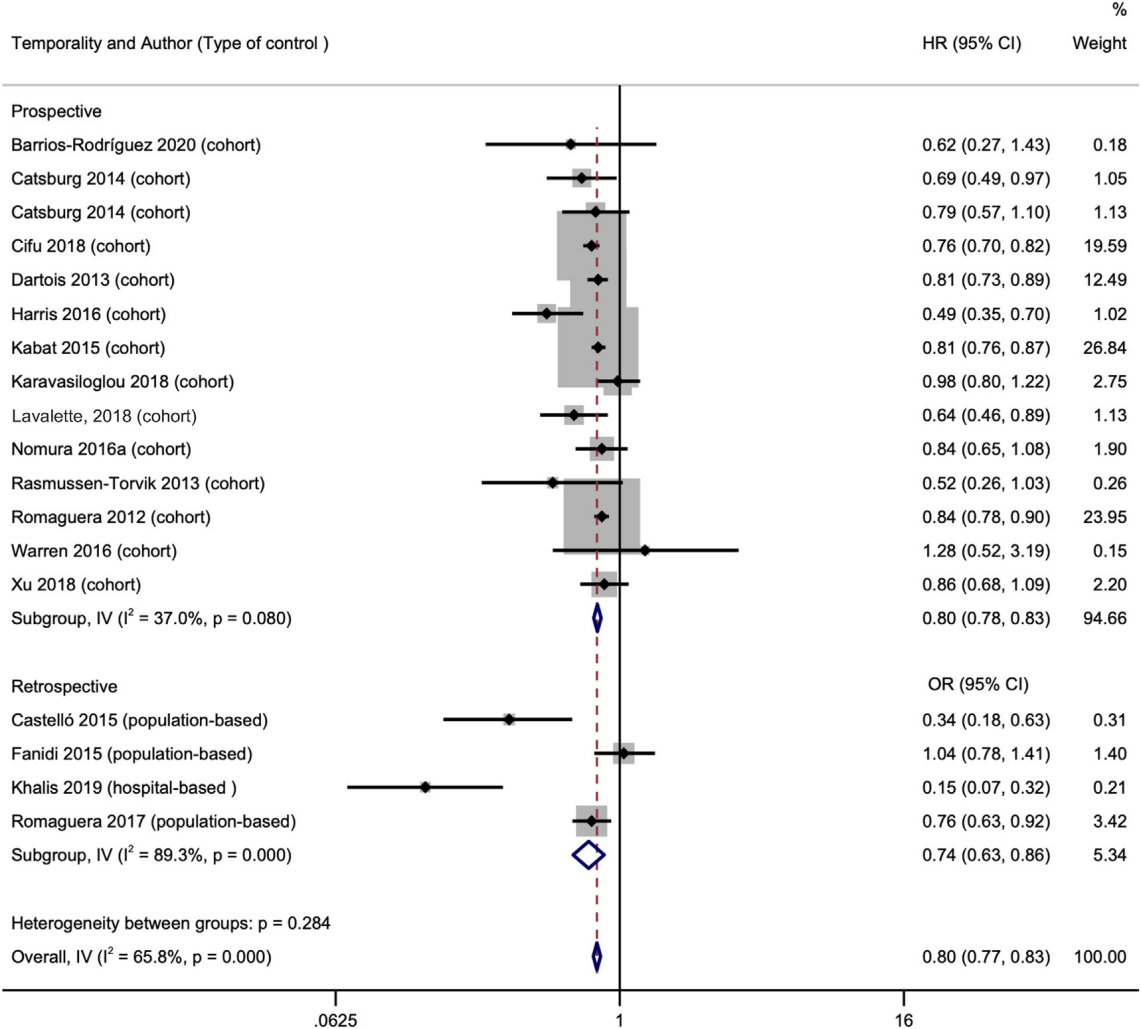
Forest plot with random effects overall hazard ratio (HR) from prospective studies and odds ratio (OR) from retrospective studies for an association between healthy lifestyle indices and BC.

For the quantitative synthesis, 2 of the 31 studies were excluded; one was not comparable with other studies [23]. Another study was excluded because it did not consider the diet variable within the index [36]. The overall HR for prospective studies was 0.80 (95% CI: 0.78, 0.83) in 14 studies with moderate certainty of the evidence, indicating that the highest adherence to a healthy lifestyle index likely reduces BC cases compared with the lowest. Similarly, for retrospective studies, the overall OR was 0.74 (95% CI: 0.63, 0.86) in 4 studies with low certainty of the evidence, which were downgraded due to high heterogeneity (Figure 3).

**FIGURE 4:**
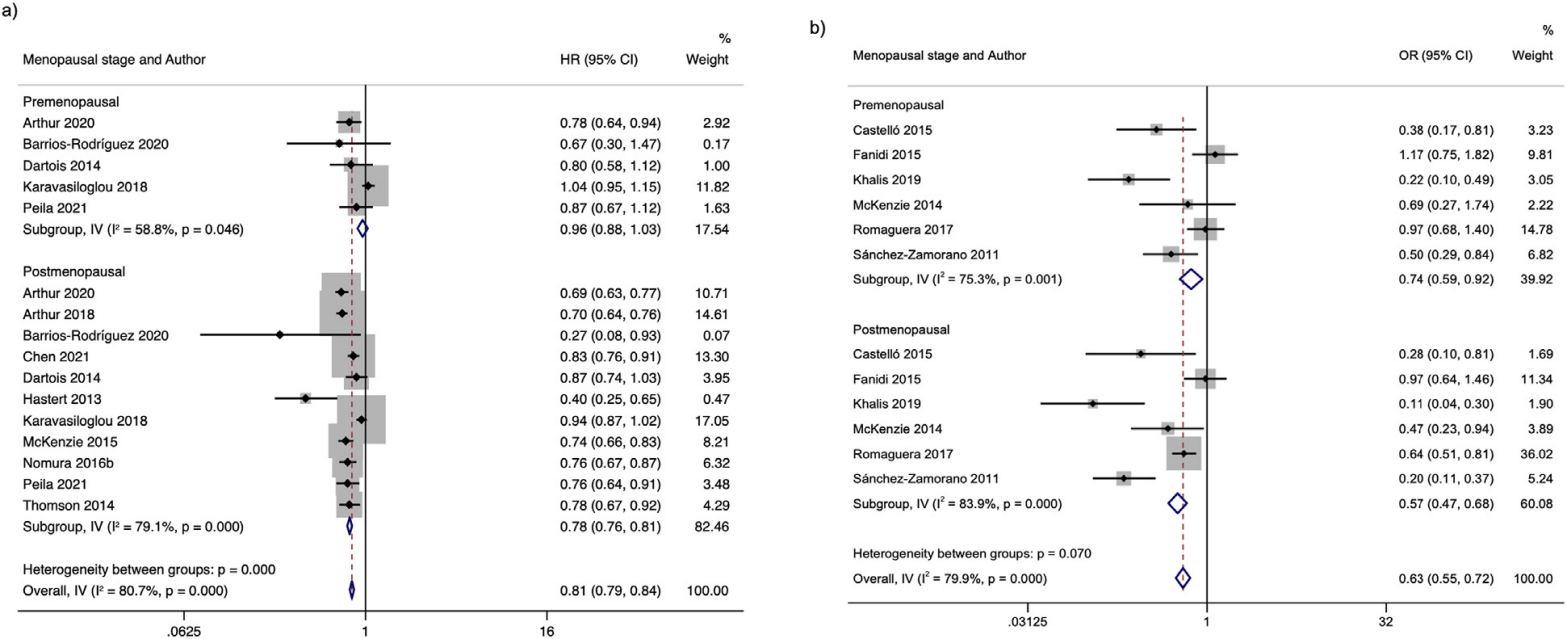
Forest plot with random effects for association between healthy lifestyle indices and BC stratified by menopausal status. a) Meta-analysis by menopausal status for prospective studies b) Meta-analysis by menopausal status for retrospective studies.

Regarding premenopausal BC, the summary HR of 5 prospective studies was 0.96 (95% CI: 0.88, 1.03), with very uncertain evidence (very low GRADE) about the association of the highest over the lowest adherence to a healthy lifestyle index and premenopausal BC. The certainty of the evidence was downgraded due to slight association and high heterogeneity, while in retrospective studies, the summary OR was 0.74 (95% CI: 0.59, 0.92) in 6 studies with moderate certainty of the evidence. Concerning postmenopausal BC, in prospective studies, an HR of 0.78 (95% CI: 0.76, 0.81) was observed for the highest adherence to a healthy lifestyle index over the lowest with moderate certainty of the evidence. This association was also observed in retrospective studies with an OR of 0.57 (95% CI: 0.47, 0.68) with high certainty of the evidence upgraded because of the high reduction of the association (Figure 4).

**FIGURE 5:**
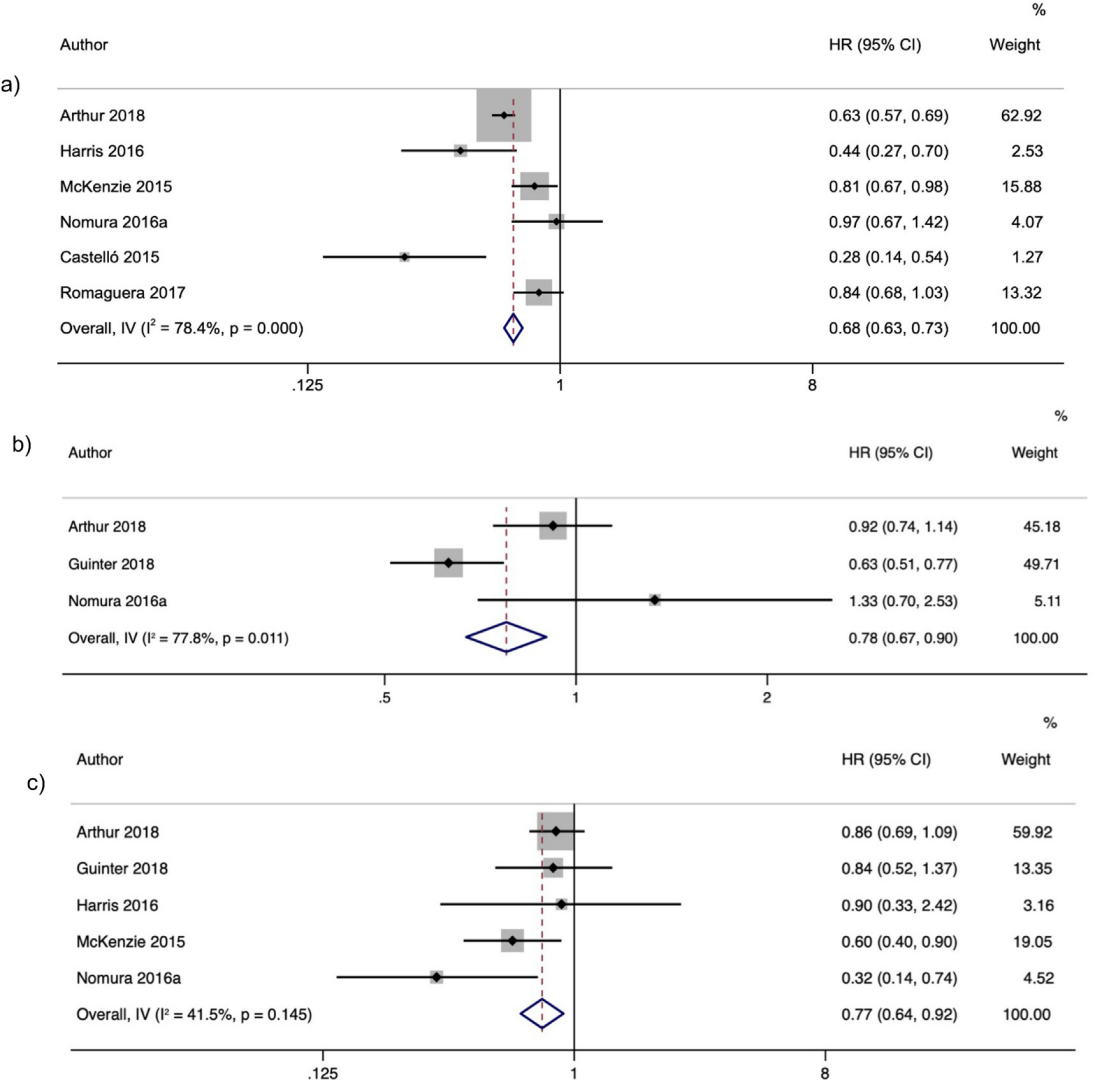
Forest plot with random effects overall hazard ratio (HR) from prospective studies and odds ratio (OR) from retrospective studies for an association between healthy lifestyle indices and BC molecular subtypes. a) Meta-analysis of ER+/PR+ BC molecular subtype (prospective and retrospective studies); b) Meta-analysis of ER+/PR- BC molecular subtype (prospective and retrospective studies); c) Meta-analysis of ER-/PR- BC molecular subtype (prospective and retrospective studies).

For molecular subtypes, the highest over the lowest compliance to a healthy lifestyle index likely reduces overall HR/OR of BC molecular subtypes: ER+/PR+(HR = 0.68; 95% CI: 0.63, 0.73), ER+/PR- (HR = 0.78; 95% CI: 0.67, 0.90), and ER-/PR- (HR = 0.77; 95% CI: 0.64, 0.92) (Figure 5); all present moderate certainty of evidence. The detailed certainty of evidence is presented in Supplemental Table 3.

### Sensitivity analysis

After performing the assessment of those studies, including a healthy lifestyle index based on WCRF/AICR or ACS guidelines for cancer prevention and those studies that represent a greater weight in the original statistical analysis, no change was observed in the overall estimate of the measure of association concerning the main analyses (Supplemental Figures 1 and 2).

### Publication bias

No publication bias was observed in the funnel plots presented in Supplemental Figures 3, 4, 5, 6, 7, and 8.

## Discussion

This study provides the most comprehensive and up-to-date summary of evidence of the association between lifestyle recommendations and BC. In this analysis, a total of 31 studies were included in the systematic review, whereas in the meta-analysis, 29 articles evaluating the association between a healthy lifestyle index and BC risk were assessed. High versus low adherence to a healthy lifestyle index was significantly associated with a 20% decrease in BC risk. Consistent reductions were also shown for postmenopausal and for molecular subtypes for those females most adherent to the WCRF/AICR and ACS lifestyle recommendations, whereas for females with premenopausal BC, the evidence was inconclusive.

The analysis carried out by epidemiological design showed a decreased risk of BC when comparing the category with the highest adherence to a healthy lifestyle index compared with the lowest, which was consistent in both prospective (HR = 0.80, 95% CI: 0.78, 0.83) and retrospective studies (OR = 0.74, 95% CI: 0.63, 0.86). The results found in the analysis of prospective studies are in line with Zhang et al., which evaluated lifestyle factors and cancer incidence, including BC. They found a 23% lower risk (95% CI: 0.72, 0.82) for the highest versus the lowest adherence to a healthy lifestyle for developing BC [26].

The results were consistent in most stratified analyses. For example, in prospective premenopausal studies, when comparing the highest versus lowest category of a healthy lifestyle index, an inverse association was observed (HR = 0.96, 95% CI: 0.88, 1.03); however, this was not significant. Whereas for postmenopausal studies, a statistically significant inverse association was observed (HR = 0.78, 95% CI: 0.76, 0.81). For retrospective studies, analyses stratified by menopausal status showed a statistically significant inverse association for premenopausal (OR = 0.74, 95% CI: 0.59, 0.92) and postmenopausal (OR = 0.57, 95% CI: 0.47, 0.68) BC comparing the highest adherence to a healthy lifestyle over the lowest.

A possible explanation for the nonstatistically significant association found in the meta-analysis stratified by premenopausal status in prospective studies is the high heterogeneity found in the variables considered as potential confounders. Although the models with the highest level of adjustment reported by the authors were considered for the results of this meta-analysis, residual confounding cannot be ruled out. Another explanation could be the small sample in these studies that included information on premenopausal BC, which may contribute to the attenuation of the association. Further, the number of incident cases was small in most of the prospective studies that found no statistically significant association for premenopausal BC [19,21,22,36], which could contribute to not having the sample size necessary to detect a statistically significant association. In addition, all prospective studies with premenopausal women include the BMI variable within the lifestyle indices, and this could be considered potentially confounding given the existing evidence of the inverse association between BMI and premenopausal BC risk [52]. Although selection bias and recall bias are likely to be present in case-control studies, in the present review, we did not identify a risk of bias in the selection domain according to the evaluation with the NOS tool among the included studies. Furthermore, we cannot rule out a recall bias related to the differential recall of dietary intake between cases and controls.

All 3 sub-analyses showed a negative association between adherence to a healthy lifestyle index and BC by molecular subtype (ER+/PR+, ER+/PR-, and ER-/PR-). Different biological mechanisms have been postulated by which lifestyle components could influence breast carcinogenesis. For example, the main biological mechanism linked to physical activity and its potential benefit in BC is that it can decrease estrogen concentrations, particularly estradiol and sex hormone-binding globulin (SHBG) [53]; increase the length of menstrual cycles; and reduce ovulation in premenopausal women with high levels of physical activity [52,54], which it could be related to BC molecular subtypes with hormone receptors. Likewise, it has been observed that alcohol consumption can increase the levels of sex hormones, such as the levels of androgens and estrogens [55]. Specifically, ethanol can stimulate cell proliferation and induce the expression of ER and PR hormone receptors [56]. Ethanol can produce lipid peroxidation and DNA damage through mechanisms of inflammation and oxidative stress [57].

The effect of tobacco consumption has been specially related to ER+BC in people with polymorphisms associated with the metabolism of tobacco compounds [54,58]. In vitro, studies have shown changes in the mammary gland exposed to cigarette smoke through changes in gene regulation, such as increased methylation of occludin and Claudin-1, as well as increased methylation of the gene that codes for the ER beta (ERβ) [59]. It has been documented that the protective association of fruits and nonstarchy vegetables could be greater in tumors that do not express hormone receptors (ER) compared to ER+ [60]. This is because phytochemicals included in foods within a healthy diet reduce levels of the Epidermal Growth Factor (EGF), which could reduce the risk of ER- BC [52]. Some of the phytochemicals present in fruits and vegetables, such as; carotenoids, glucosinolates, indoles, and isothiocyanates, could reduce the risk of developing BC due to the activity of detoxifying enzymes, which can reduce oxidative stress and inflammation and modify the epigenome [7].

Our study has some important limitations. First, all analyses showed substantial heterogeneity. This can be explained by the great diversity of the types of lifestyle indices included in the studies since not all of them considered the same number or type of variables, although many indices are based on adherence to recognized or standardized guidelines for cancer prevention, such as those from the WCRF/AICS or the ACS. Other studies used a combination of these and other cancer prevention guidelines for the construction of lifestyle indices. In addition, even when some indices could coincide with the variables that compose them, the number of variables and operationalization of these variables were different in most of the studies. Another possible explanation for the high heterogeneity observed is that each one of the healthy lifestyle components probably has a different weight in each population, even though they are being analyzed together as an index. Despite the heterogeneity observed in the different sub-analyses, the low risk of bias found in most of the studies included in this review might indicate their internal validity. In addition, although we summarized the results of models with the highest level of adjustment, residual confounding cannot be ruled out. Moreover, in prospective studies, components of the adherence score were measured singularly at baseline and used to assess BC risk over time. Repeated measurements of lifestyle variables may have provided an improved exposure assessment of long-term behavior and risk over time. Additionally, follow-up times ranged from 5 to 23 y (with most cases less than 10 y), which may not be sufficient for assessing the protective role of adherence to lifestyle factors and BC prevention. However, observational studies are the most appropriate to evaluate this type of exposure and outcomes since it is difficult to find an RCT with enough time for follow-up and to identify cancer as an outcome and lifestyle interventions; also, in our pilot search, we could not identify any RCT that include a healthy lifestyle index or interventions on more than one of the components of the healthy lifestyle (diet or physical activity), and BC as an outcome. Finally, no publication bias was found, reflecting the representativeness of the studies included in this meta-analysis, so it is unlikely to substantially alter the overall findings of this study.

Our study has some strengths. The risk of the bias assessment tool is validated for observational studies, and this assessment was stratified by study design. Additionally, in the GRADE evaluation, most of the sub-analyses obtained a rating of moderate to high certainty of evidence.

In conclusion, to our knowledge, this is the first systematic review of observational studies regarding healthy lifestyle indices and BC that explores the relationship between BC molecular subtype and menopausal status. Adherence to a healthy lifestyle (a healthy diet, moderate-vigorous intensity physical activity, low alcohol consumption, low tobacco consumption, and breastfeeding) may reduce the risk of BC in general, postmenopausal BC, and BC by ER+/PR+, ER+/PR-, and ER-/PR- molecular subtype. These findings should be considered to generate recommendations for BC prevention at the population level, considering the specific characteristics of each population.

### Differences between protocol and review

The protocol was first registered in PROSPERO in August 2021 before starting the review. After the final pilot test, the research team, in consensus, decided to use the NOS tool to evaluate the risk of bias in the included studies instead of the risk of bias in the nonrandomized studies (ROBINS-I) tool. According to the COSMOS-E guide, this tool is the one that would work better for the type of epidemiological designs included in our systematic review. The NOS tool is validated and recommended for cohorts and case-control studies.

### Funding

Supported by National Council for Science and Technology (CONACyT by its acronym in Spanish) and Sector Fund for Research in Health and Social Security (FOSISS) (SALUD-2005-C02-14373).

### Author disclosures

The authors declare no conflict of interest.

### Acknowledgments

The authors’ responsibilities were as follows—BIAG, AGR, EDG: designed research; BIAG, AGR, EDG, LLC: conducted research; BIAG, AGR: analyzed data; BIAG, AGR, EDG, LLC, AMO: wrote the paper; EDG, BIAG: had primary responsibility for the final content. All authors have read and approved the final manuscript.

### Data availability

The datasets generated during and/or analyzed during the current study are available from the corresponding author upon reasonable request.

## Appendix A Supplementary data

Multimedia component1 Multimedia component1
