# Supplementary material for: Lifestyle Quality Indices and Female Breast Cancer Risk: A Systematic Review and Meta-Analysis
Source: Adv Nutr. 2023 Apr 20;14(4):685–709. doi: 10.1016/j.advnut.2023.04.007 (PMC10334144; doi:10.1016/j.advnut.2023.04.007)
Supplement: Multimedia component1 [file mmc1.docx]

**Title:** Association between lifestyle quality indices and breast cancer risk in adult women: a Systematic Review and Meta-analysis.

**Brianda I. Armenta-Guirado^1,2^, Alejandra González-Rocha^1^, Ángel Mérida Ortega^3^, Lizbeth López-Carrillo^3^, Edgar Denova-Gutiérrez^1^**

| **Supplemental Table 1.** Search algorithm for the systematic review of lifestyle quality indices and breast cancer | | |
| --- | --- | --- |
| **Databases** | **Search algorithm** | **Time limits** |
| **PubMed** | (healthy lifestyle index) AND ((breast neoplasms[MeSH Terms]) OR (breast cancer)) | 2000-2022 |
|  | (life style[MeSH Terms]) AND ((breast neoplasms[MeSH Terms]) OR (breast cancer)) | 2000-2022 |
| **Lilacs** | (healthy lifestyle index) AND ((breast neoplasms) OR (breast cancer)) AND (year_cluster:[2000 TO 2021]) | 2000-2022 |
|  | (life style) AND ((breast neoplasms) OR (breast cancer)) AND ( mj:("Estilo de Vida" OR "Neoplasias de la Mama") AND la:("en" OR "es")) AND (year_cluster:[2000 TO 2021]) | 2000-2021 |
| **CINAHL** | healthy lifestyle index AND (breast neoplasms or breast cancer or breast tumor) | 2000-2022 |
|  | lifestyle index AND (breast cancer or breast neoplasm or breast carcinoma or breast tumor) | 2000-2022 |
| **ScienceDirect** | (healthy lifestyle index) AND ((breast neoplasms) OR (breast cancer)) | 2000-2022 |

| **Supplemental Table 2.** Methodological quality (risk of bias) assessment with The Newcastle-Ottawa Scale (NOS) tool of prospective and retrospective studies included in systematic review of lifestyle quality indices and breast cancer. | | | | | | | | | |
| --- | --- | --- | --- | --- | --- | --- | --- | --- | --- |
|  | **Selection** | | | | **Comparability** | **Outcome** | | |  |
| **Quality assessment of prospective^a^ studies** | | | | | | | | | |
| **Study** | **Representativeness of the exposed cohort** | **Selection of the non-exposed cohort** | **Ascertainment of exposure** | **Demonstration that outcome of interest was not present at start of study** | **Comparability of cohorts on the basis of the design or analysis controlled for confounders** | **Assessment of outcome** | **Was follow-up long enough for outcomes to occur** | **Adequacy of follow-up of cohorts** | **Quality score** |
| Arthur et al. 2018 | Consecutive or obviously representative series of cases  *1936 postmenopausal*  *Sub cohort of Canadian Study of Diet, Lifestyle and Health*  *410 incident cases* | Drawn from the same community as the exposed cohort | At least one questionnaire ascertainment of exposure validated  *Self-administered quantitative* *food frequency questionnaire (FFQ) and a lifestyle questionnaire. FFQ was calibrated* | Yes | Study controls for the most important factor and additional factors  *The study controls for age, sex and marital status. Adjusted for education, non-alcohol energy intake, smoking status, alcohol intake, BMI, diet score, physical activity, age at menarche, parity, breastfeeding,* *HRT use, oral contraceptive use, family history* | Record linkage  *Incident cancer cases were ascertained via record linkage to the* *Canadian Cancer Registry (CCR) and to the Ontario Cancer Registry* | No  *5 years follow up* | Complete follow up- all subject accounted for | Good  ★★★★★★★★ |
|  | ★ | ★ | ★ | ★ | ★★ | ★ |  | ★ |  |
| Arthur et al. 2018 | Truly representative  *Postmenopausal women ages 50 to 79, from major racial/ethnic groups, who were enrolled at 40 clinical centers through- out the United States between 1993 and 1998. 31,833 (n 1⁄4 84,476 and n= 47,357 from the* *observational study (OS) and clinical trial (CT), respectively) postmenopausal women who were followed up until September 30, 2016* | Drawn from the same community as the exposed cohort | Not validated  *The reliability of the FFQ was assessed by calculating intraclass correlation coefficients between the first and second administration of the questionnaire* | Yes | Study controls for the most important factor and additional factors  *The study controls for age and marital status. Adjusted for age at entry, education, non-alcohol energy intake, ethnicity, age at menarche, parity, breastfeed, history of mammograms, HT status, oral contraceptive use, age at menopause, family history, history of BBD, diet, alcohol intake, physical activity, and BMI smoking unless included as main exposure* | Record linkage  *Breast cancer cases were confirmed centrally by trained physician adjudicators who reviewed medical records and pathology reports.*  *Coding of breast cancer characteristics (tumor hormone receptor status, histology, nodal involvement, grade, and stage) was performed using the NCI's Surveillance Epidemiology and End Results coding system* | Yes  *18 years follow up* | Complete follow up- all subject accounted for | Good  ★★★★★★★★ |
|  | ★ | ★ |  | ★ | ★★ | ★ | ★ | ★ |  |
| Arthur et al, 2020 | Somewhat representative  *UK Biobank is a large prospective observational study comprising approximately 500000 men and women (N=229 134 men, N=273 402 women) of various ethnicities aged 40–69 years at enrollment. Participants were recruited from across 22 centers located throughout England, Wales, and Scotland between 2006 and 2010. The study was established to provide a resource for the investigation of the genetic, environmental, and lifestyle factors associated with a wide range of diseases, including cancer* | Drawn from the same community as the exposed cohort | At least one questionnaire ascertainment of  *exposure validated*  *UK Biobank food frequency questionnaire* | Yes  *The outcome for this study was incident invasive breast cancer. In UK Biobank, cancer diagnoses were ascertained through link- age to national cancer registries in England, Wales, and Scotland.* | Study controls for the most important factor and additional factors  *The study controls for age, sex and marital status. Adjusted for age at recruitment, socioeconomic status, age at menarche, parity and age at first pregnancy, family history of breast cancer, history of mammograms, oral contraceptive use, age at menopause, hormone replacement therapy use, body mass index (premenopausal women)* | Record linkage  *In UK Biobank, cancer diagnoses were ascertained through linkage to national cancer registries in England, Wales, and Scotland* | No  *Follow-up of 7.1 years* | Complete follow up- all subject accounted for | Good  ★★★★★★★★ |
|  | ★ | ★ | ★ | ★ | ★★ | ★ |  | ★ |  |
| Barrios-Rodríguez 2020 | Truly representative  *SUN (“Seguimiento Universidad de Navarra”) prospective cohort* | Drawn from the same community as the exposed cohort | At least one questionnaire ascertainment of exposure validated  *Physical activity was ascertained at baseline through a validated 17-item questionnaire*  *Previously validated 136-item food-frequency questionnaire (FFQ). Further, the reproducibility of the FFQ was specifically addressed in a subsample of this cohort* | Yes | Study controls for the most important factor and additional factors  *The study controls for age, years at university, smoke habits, family history of breast cancer, age of menopause. Adjusted for age at recruitment, socioeconomic status [based on Townsend deprivation index as quintiles, age at menarche, parity and age at first live birth (as a combined variable), ever use of hormone replacement therapy (HRT; post- menopausal women only), ever use of oral contraceptives, his- tory of mammograms, age at menopause* | Evaluated by a trained oncologist who was blinded to exposures of participants and adjudicated the confirmed cases. | Yes  *16-year and 18-year follow-up* | Complete follow up - all subjects accounted for | Good  ★★★★★★★★★ |
|  | ★ | ★ | ★ | ★ | ★★ | ★ | ★ | ★ |  |
| Catsburg, 2014 | Truly representative  *Women participating in the* *Canadian National Breast Screening Study (NBSS). The NBSS was a randomized, controlled trial of breast cancer screening. The NBSS recruited 89,835 women, aged 40–59, from 15 Canadian clinical centers between 1980 and 1985.* | Drawn from the same community as the exposed cohort  *Randomization of the CT NCBSS* | At least one questionnaire ascertainment of exposure validated  *A modified version of a previously validated, self-administered food frequency questionnaire (FFQ) was distributed to all new attendees at screening centers and also to women returning to the screening centers for rescreening.* | Yes  *Cases in our study were women diagnosed with incident, invasive breast cancer during follow-up in the NBSS, ascertained by means of computerized record linkage to the* *Canadian Cancer Registry (CCR)* | Study controls for the most important factor and additional factors  *Age, age at menarche, use of oral contraceptives, use of hormone therapy, age at first live birth, family history of breast cancer, history of breast disease, menopausal status at baseline, and study center.* | Record linkage  *Invasive breast cancer during follow-up in the NBSS, ascertained by means of computerized record linkage to the CCR. In addition, cancer diagnosis and histology are confirmed using pathology reports, thereby ensuring that breast cancer cases are correctly classified.* | Yes  *Follow-up of 16.6 years* | Complete follow up- all subject accounted for | Good  ★★★★★★★★★ |
|  | ★ | ★ | ★ | ★ | ★★ | ★ | ★ | ★ |  |
| Chen, 2021 | Truly representative  *NOWAC study is a nationwide, prospective cohort study, consisting of approximately 172,000 adult female participants.* | Drawn from the same community as the exposed cohort  *Women invited to participate in the NOWAC study were randomly sampled from the Norwegian Central Person Register between 1991 and 2007 in multiple sub-cohort* | At least one questionnaire ascertainment of exposure validated  *Physical activity level, BMI, and the FFQ have been vali- dated in the NOWAC study.* | Yes  *Women with prevalent cancer, those who died or emigrated before baseline, and those with extreme energy intakes (<2100 or >15,000 KJ/day) were excluded, leaving 96,869 cancer-free participants in the final study sample* | Study controls for the most important factor and additional factors  *Education, height, age at menarche, use of oral contraceptives, parity, breastfeeding, use of hormone replacement therapy, family history of breast cancer in a first-degree relative.* | Record linkage  *Women diagnosed with incident cancer after baseline were identified through linkage to the Cancer Registry of Norway, based on codes from the International Classification of Diseases for Oncology* | Yes  *A median follow-up of 20.0 years* | Complete follow up- all subject accounted for | Good  ★★★★★★★★★ |
|  | ★ | ★ | ★ | ★ | ★★ | ★ | ★ | ★ |  |
| Cifu et al, 2018 | Somewhat representative  *AARP members, aged 50-71 years, from six different states*  *From six different states (California, Florida, Pennsylvania, New Jersey, North Carolina, and Louisiana) and two metropolitan areas (Atlanta and Detroit) with state cancer registries.* | Drawn from the same community as the exposed cohort | At least one questionnaire ascertainment of exposure validated  *The food frequency questionnaire completed by NIH-AARP Diet and Health Study participants was validated in a sub-cohort* | Yes | Study controls for the most important factor and additional factors  *Adjusted by models*  *Model 1: HRs adjusted for hormone therapy use, education status, history of a first-degree relative with breast cancer, and general health status. Model 2: HRs adjusted for lag time between cohort entry and diagnosis. Model 3: HRs adjusted for hormone therapy use, education status, history of a first-degree relative with breast cancer, general health status, grade, stage, and lag time*. | Cases of incident breast cancer were identified by linkage  *Between the NIH-AARP cohort and state cancer registries.* | Yes  *The mean follow-up time was 12.5 (±4.4) years.* | Complete follow up - all subjects accounted for  *All incident primary breast cancer cases identified through the study follow-up in 2011 were included in our analysis.* | Good  ★★★★★★★★★ |
|  | ★ | ★ | ★ | ★ | ★★ | ★ | ★ | ★ |  |
| Dartois et al, 2013 | Truly representative of the average in the community  *64,732 women ages 43 to 68 years at baseline. The E3N [Etude Epidemiologique aupres des femmes de la Mutuelle Générale de l'Education Nationale (MGEN)] to investigate cancer risk factors* | Drawn from the same community as the exposed cohort | No description  *Women were asked to fill in a dietary questionnaire using quantitative and qualitative estimates of consumed items, including alcohol consumption and fruit and vegetable consumption. Validation studies, conducted to determine the accuracy of the reported anthropometric measurements and dietary data, demonstrated the reliability of the reported data* | Yes  *Participants with breast cancer, both confirmed and probable cases, were followed up until the date of breast cancer diagnosis, and those without breast cancer until the date of death or last contact.* | Study controls for the most important factor and additional factors  *The study control for sex, age, menopausal stage.* *Adjusted for level of education, residence, first-degree family history of any cancer, professional activity, use of oral contraceptives, age at menarche and number of children, age at first full-term pregnancy, menopausal status, and use of menopausal hormone therapy. Adjusted for level of education, residence, first-degree family history of any cancer, professional activity, use of oral contraceptives, age at menarche and number of children, and age at first full-term pregnancy. Adjusted for level of education, residence, first-degree family history of any cancer, professional activity, use of oral contraceptives, age at menarche and number of children, age at first full-term pregnancy, and use of menopausal hormone.* | Unclear  Self-report  *All questionnaires enquired about occurrence of any cancer, type of cancer, addresses of physicians, and permission to contact them. Invasive cancer cases were confirmed by pathology reports or death certificates, obtained for 86.1% of our population cases* | Yes  *Median follow-up of 8 years for invasive cancer cases and 15 years for non-cases* | Unclear | Fair  ★★★★★★ |
|  | ★ | ★ | ★ | ★ | ★★ |  | ★ |  |  |
| Guinter et al, 2018 | Truly representative of the average women in risk of cancer  *27,153 women enrolled in the Prostate, Lung, Colorectal, and Ovarian Cancer Screening Trial*. | Drawn from the same community as the exposed cohort | At least one questionnaire ascertainment of exposure validated  *Dietary data were collected via the DQX, a 137-item food frequency questionnaire (FFQ) designed specifically for PLCO to assess typical frequency of intake over the past year* | Yes | Study controls for the most important factor and additional factors  *Adjustment for age, TEI, PMH, education, BMI at age 20, bilateral oophorectomy, parity, age at menopause, family history of breast cancer, race/ethnicity, and study center* | Record linkage  *Over 96% of PLCO cases were confirmed through hospital records* | Yes  *Median follow-up of 11.5 years* | Complete follow up - all subjects accounted for | Good  ★★★★★★★★ |
|  | ★ | ★ |  | ★ | ★★ | ★ | ★ | ★ |  |
| Harris et al, 2016 | Truly representative of the average women in central Sweden.  *31,514 Swedish Mammography Cohort (SMC)* | Drawn from the same community as the exposed cohort | At least one questionnaire ascertainment of exposure validated  *The physical activity questions have been previously validated and have been shown to correlate well with total physical activity.*  *The FFQ has been previously validated.* | Yes | Study controls for the most important factor and additional factors  *Age, height, education, oral contraceptive use, hormone replacement therapy use, age at menarche, menopausal status/age at menopause, family history of breast cancer, history of benign breast disease and smoking status.* | Record linkage  *Histologically confirmed incident invasive breast cancer case* | Yes  *15 years follow-up* | Complete follow up - all subjects accounted for | Good ★★★★★★★★★ |
|  | ★ | ★ | ★ | ★ | ★★ | ★ | ★ | ★ |  |
| Hastert et al, 2013 | Truly representative of the average women in Washington.  *30,797 women. The Vitamins and Lifestyle (VITAL) study is a prospective cohort study designed to investigate the associations between use of dietary supplements and cancer risk. 13 counties in the Western Washington Surveillance, Epidemiology and End Results (SEER) cancer registry at baseline*. | Drawn from the same community as the exposed cohort | At least one questionnaire ascertainment of exposure validated  *Validity studies of similar FFQs have reported correlation* | Yes | Study controls for the most important factor and additional factors  *Sex, age, BMI. Adjusted for age (as the timeline in the Cox model), education, race, mammography, family history of breast cancer, age at menarche, age at first birth, age at menopause, and years of estrogen plus progestin hormone therapy use using categories in Table 2 and daily energy intake (kcal). Models included 30,797 observations and 899 incident breast cancers.* | Record linkage  *Linkage between VITAL and SEER is largely automated and based on ranking agreement between items common. All incident cancers diagnosed in the 13 counties of the Western Washington SEER registry (except for nonmelanoma skin cancers) are reported to SEER by all area hospitals and by offices of pathologists, oncologists, and radiotherapists*. | No  *6.7 years follow-up* | Complete follow up - all subjects accounted for | Good ★★★★★★★★ |
|  | ★ | ★ | ★ | ★ | ★★ | ★ |  | ★ |  |
| Kabat, 2015 | Truly representative  *The NIH-AARP Diet and Health Study (www.clinicaltrials. gov; NCT00340015) is a large prospective cohort study of AARP members initiated in 1995–1996.* | Drawn from the same community as the exposed cohort  *Members between the ages of 50 and 71 y who resided in 6 states (California, Florida, Louisiana, New Jersey, North Carolina, or Pennsylvania) or in 2 metropolitan areas (Atlanta, or Detroit) with existing population-based cancer registries.* | No description | Yes | Study controls for the most important factor and additional factors  *Age, educational level, ethnicity, smoking status, marital status, and energy intake. Breast, ovarian, and endometrial cancers also were adjusted for menopausal status, age at menarche, age at first birth, parity, and hormone therapy use. Breast cancer also was adjusted for family history of breast cancer in a first-degree relative and mammographic screening* | Record linkage  *Incident cases were identified from cancer registries in the original 6 states and 2 metropolitan areas, plus Texas and Arizona, states to which participants most commonly moved during follow-up. A validation study indicated that study procedures identified w 90% of all incident cancers within the 8 registries. Areas with high quality cancer registry* | Yes  *A median follow-up of 10.5 y* | Complete follow up - all subjects accounted for | Good ★★★★★★★★ |
|  | ★ | ★ |  | ★ | ★★ | ★ | ★ | ★ |  |
| Karavasiloglou et al, 2018 | Truly representative of the average women in Europe, between 20-70 years.  *EPIC is a multicenter prospective cohort study*  *260,151 women* | Drawn from the same community as the exposed cohort | At least one questionnaire ascertainment of exposure validated  *12 months was assessed using validated country-specific questionnaires* | Yes  *Women who developed first primary incident and histologically confirmed BCIS between recruitment and the latest date of complete information were considered as cases.* | Study controls for the most important factor and additional factors  *Model adjusted for the highest level of attained education, smoking status, total energy intake, presence of chronic diseases at recruitment, age at menarche, age at first full-term pregnancy, menopausal status, ever use of oral contraceptive pills, and ever use of menopausal hormone therapy. Additionally, all individual components were adjusted for the remaining components of the WCRF/AICR lifestyle score. All analyses were stratified for center and age at recruitment (1-year intervals).* | Record linkage  *Health insurance records, cancer pathology registries, and active follow-up of study participants and their next of kin. Women who developed first primary incident and histologically confirmed BCIS between recruitment and the latest date of complete information were considered as cases.* | Yes  *14.9 follow-up years* | Complete follow up - all subjects accounted for | Good ★★★★★★★★★ |
|  | ★ | ★ | ★ | ★ | ★★ | ★ | ★ | ★ |  |
| Lavalatte et al, 2018 | Somewhat representative of the average French women in the community.  *The NutriNet-Sante study is a French ongoing web-based cohort launched in 2009 with the objective to investigate the associations between nutrition and health as well as the determinants of dietary behaviors and nutritional status*. | Drawn from the same community as the exposed cohort | At least one questionnaire ascertainment of exposure validated  *All questionnaires are completed online using a dedicated website. Portion sizes were estimated using validated photographs* | Yes | Study controls for the most important factor and additional factors  *Sex, age. Adjusted for age, height, smoking status, number of dietary records, energy intake without alcohol, family history of cancer among first-degree relatives, higher education, body mass index and physical activity. Adjustment for BMI and physical activity were not performed for scores in which they were included as components* | Written self-report  *Participants were contacted by a physician of the team and asked to provide any relevant medical records. Not histological pathology.*  *Medical records were obtained for >90% of cancer cases. Because of the high validity of self-reports (95% of self- reported cancers for which a medical record was obtained were confirmed by our physicians), we included as cases all participants who self-reported incident cancers, unless they were identified as non-cases participants by a pathology report, in which case we classified them as non-case* | No  *8 years follow-up* | Complete follow up - all subjects accounted for | Good  ★★★★★★★★ |
|  | ★ | ★ | ★ | ★ | ★★ | ★ |  | ★ |  |
| Lofterød et al, 2020 | Truly representative of the average women in Norway over 20 years.  *Energy Balance and Breast Cancer Aspects throughout life (EBBA-Life) study. Sub-study of the Tromsø study.* | Drawn from the same community as the exposed cohort | No description  *The questionnaires were filled in at home and brought to the study site.* *Questionnaires included items about medical history, specific symptoms, dietary habits, lifestyle factors, reproductive factors, and use of medication including anti- hypertensive drugs and hormone therapy* | Yes | Study controls for the most important factor and additional factors  *Sex, age. Adjusted for age and number of live births* | Record linkage  *Cancer Registry of Norway by using the unique, national, 11-digit identification number* | No  *9.1 years follow up* | Complete follow up - all subjects accounted for | Good  ★★★★★★★ |
|  | ★ | ★ |  | ★ | ★★ | ★ |  | ★ |  |
| McKenzie, et al 2015 | Truly representative of the average post-menopausal women in Europe, between 25-70 years.  *242,918 postmenopausal women. EPIC is a multicenter prospective cohort study. 23 centers in 10 European countries (Denmark, France, Germany, Greece, Italy, the Netherlands, Norway, Spain, Sweden and the United Kingdom)* | Drawn from the same community as the exposed cohort | At least one questionnaire ascertainment of exposure validated  *12 months was assessed using validated country-specific questionnaires*  *In an* $\sim$*8% random sub-sample, standardized interviewer-administered 24-hr recalls were collected in a calibration study.* | Yes | Study controls for the most important factor and additional factors  *Age. Models were adjusted by height, age at menarch, age at full term pregnancy, education. oral contraceptive use, hormone replacement therapy use, breastfeeding, total energy intake excluding alcohol.* | Record linkage  *Health insurance records, cancer pathology registries, and active follow-up of study participants and their next of kin.* | Yes  *10.9 follow-up years* | Complete follow up - all subjects accounted for | Good  ★★★★★★★★★ |
|  | ★ | ★ | ★ | ★ | ★★ | ★ | ★ | ★ |  |
| Nomura et al, 2016 | Truly representative of the average north American black women between 21-69 years.  *The Black Women’s Health Study (BWHS)* | Drawn from the same community as the exposed cohort | At least one questionnaire ascertainment of exposure validated  *The baseline FFQ was previously validated in this*  *A validation study of self-reported physical activity and body measures was previously published* | Yes | Study controls for the most important factor and additional factors  *Age, smoking status, education, hormone replacement therapy usage. Additional covariates included family history of breast cancer, menarche ag, menopause age, parity except in models where associations were evaluated according to that non-modifiable risk factor. Similarly, BMI/alcohol/physical activity variables were included in models where BMI/alcohol/physical activity score were not the exposure of interest.* | Self-reported  *Cases of invasive breast cancer from baseline through 2011 were ascertained through self-report on the follow-up questionnaires and through linkage with 24 cancers registries in states in which 95% of participants lived.*  *Hospital or registry pathology data were obtained for >85% of cases* | Yes  *13.86 y follow-up* | Complete follow up - all subjects accounted for | Good  ★★★★★★★★ |
|  | ★ | ★ | ★ | ★ | ★★ |  | ★ | ★ |  |
| Nomura et al, 2016 | Truly representative of the average postmenopausal women between 55-69 years.  *Iowa Women’s Health Study* | Drawn from the same community as the exposed cohort | At least one questionnaire ascertainment of exposure validated  *Harvard FFQ. This questionnaire has been validated in the IWHS population.* | Yes | Study controls for the most important factor and additional factors  *Age. Unadjusted and adjusted for age, smoking, education, hormone replacement therapy, family history of breast cancer, menarche age, menopause age, and parity. Age at menopause – age at menarche 5 years of potential fertility.* | Record linkage  *State Health. Registry of Iowa, a member of the National Cancer Institute’s Surveillance, Epidemiology and End Results (SEER) program*. | Yes  *23 y follow-up* | Complete follow up - all subjects accounted for | Good  ★★★★★★★★★ |
|  | ★ | ★ | ★ | ★ | ★★ | ★ | ★ | ★ |  |
| Peila, 2021 | Truly representative of the average community  *UK Biobank is a prospective cohort study including approximately 500,000 individuals (54.4% women) aged 40–69 years at recruitment, living in England, Wales and Scotland, and registered with the United Kingdom’s National Health Service (NHS)* | Drawn from the same community as the exposed cohort | At least one questionnaire ascertainment of exposure validated  *FFQ validated and R24H* | Yes  *The present study included women with normal BMI (18.5-<25.0 kg/m2) (n=105,680), who were breast cancer-free at baseline (excluded prevalent cases, n=3,100)* | Study controls for the most important factor  *Age at enrollment. socioeconomic status, race, height, family history of breast cancer, use of hormone replacement therapy, use of oral contraceptive, number of live births, history of mammogram screening, and age at menopause.* | Record linkage  *Incident invasive breast cancer cases were ascertained through the Health & Social Care Information Centre for women resident in England and Wales, and the NHS in Scotland. Code C50 was used to identify cases of invasive breast cancer based on the International Classification of Diseases, Tenth Revision (ICD-10).* | No  *An average follow-up period of 7.0* | Complete follow up - all subjects accounted for | Good ★★★★★★★★ |
|  | ★ | ★ | ★ | ★ | ★★ | ★ |  | ★ |  |
| Rasmussen, 2013 | Truly representative of the average *community*  *4 communities: Forsyth County, North Carolina; Jackson, Mississippi; suburban areas of Minneapolis, Minnesota; and Washington County, Maryland.* | Drawn from the same community as the exposed cohort | At least one questionnaire ascertainment of exposure validated  *FFQ validated* | No description | Age, race, and ARIC center | Record linkage  *Incident cancer cases from 1987 to 2006 were obtained by linking to cancer registries.* | Yes  ≈*10 years of follow-up* | Complete follow up - all subjects accounted for | Good ★★★★★★★ |
|  | ★ | ★ | ★ |  | ★ | ★ | ★ | ★ |  |
| Romaguera et al, 2012 | Truly representative of the average women in Europe, between 25-70 years.  *260,098 women. EPIC is a multicenter prospective cohort study*. 23 centers in 10 European countries | Drawn from the same community as the exposed cohort | At least one questionnaire ascertainment of exposure validated  *12 months was assessed using validated country-specific questionnaires* | Yes | Study controls for the most important factor and additional factors  *Educational level, presence of chronic diseases at baseline, smoking status and intensity of smoking, menopausal status, ever use of hormone replacement therapy, ever use of contraception pills, age at menarche, parity, age at first full-time pregnancy, and total energy.* | Record linkage  *Health insurance records, cancer pathology registries, and active follow-up of study participants and their next of kin.* | Yes  *11.0 years* | Complete follow up - all subjects accounted for | Good  ★★★★★★★★★ |
|  | ★ | ★ | ★ | ★ | ★★ | ★ | ★ | ★ |  |
| Thomson et al., 2014 | Truly representative of the average women in United States between 50-79 years.  *40 U.S. clinical centers*  *65,838 women of Women’s Health Initiative Observational Study.* | Drawn from the same community as the exposed cohort | At least one questionnaire ascertainment of exposure validated  *WHI food frequency questionnaire (FFQ).* | Yes | Study controls for the most important factor and additional factors  *Age, BMI. Adjusted for age, education, NSAID use baseline, aspirin use baseline, unopposed estrogen use, estrogen þ progestin use, multivitamin use, race/ethnicity, energy intake, parous, mammogram, family history of cancer, and having a current healthcare provider.* | Self-reported  *Medical records were collected for verification of self-reported study outcomes (cancer and death) and reviewed by trained WHI outcomes adjudicators.* | Yes  *12.6 years* | Complete follow up - all subjects accounted for | Good  ★★★★★★★★ |
|  | ★ | ★ | ★ | ★ | ★★ |  | ★ | ★ |  |
| Warren, 2016 | Truly representative of the average women from the Southern Community  *Data available for analysis are from the Southern Community Cohort Study (SCCS), a previously-described prospective cohort study conducted in 12 southeastern U.S. states that enrolled nearly 85,000 participants from 2002–2009* | Drawn from the same community as the exposed cohort  *Participants were primarily recruited from community health centers (CHC; 86%), which provide health services to medically underserved populations* | At least one questionnaire ascertainment of exposure validated  *Dietary intake was evaluated by an 89-item FFQ, developed, and validated specifically for the diet in the southeastern United States* | Yes  *For our analysis, we included only invasive cancers* | Study controls for additional factors  *Race, enrollment source, family history of cancer, insurance coverage, education, income, marital status, neighborhood deprivation index, smoking status, total energy intake, and postmenopausal hormone use and for menopausal status.* | Record linkage  *Ascertainment of incident cancer diagnoses was carried out via linkage to the 12 state cancer registries in the study area (Alabama, Arkansas, Florida, Georgia, Kentucky, Louisiana, Mississippi, North Carolina, South Carolina, Tennessee, Virginia, and West Virginia)* | No  *A median follow-up time of 6 years (range: 2–10 years)* | Complete follow up - all subjects accounted for | Good ★★★★★★★ |
|  | ★ | ★ | ★ | ★ | ★ | ★ |  | ★ |  |
| Xu, 2018 | Truly representative  *From 2001 to 2009, 31 208 adults living in Alberta, Canada were enrolled into ATP by random digit dialing, which facilitated balanced recruitment across the province.* | Drawn from the same community as the exposed cohort | At least one questionnaire ascertainment of exposure validated  *Canadian Diet History* Questionnaire (C-DHQ) | Yes  *With no previous cancer diagnosis were included in analyses.* | Study controls for the most important factor and additional factors  *Age, sex, marital status, education level, employment status, annual household income, tobacco exposure, first-degree family history of cancer and personal history of chronic disease, as well as hormone replacement therapy* | Record linkage  *The primary outcome in the present study was any malignant cancer incidence identified via linkage with the ACR.* | Yes  *A mean follow-up of 11.7* | Complete follow up - all subjects accounted for | Good  ★★★★★★★★ |
|  | ★ | ★ | ★ | ★ | ★★ | ★ | ★ | ★ |  |

|  | **Selection** | | | | **Comparability** | **Outcome** | |  |
| --- | --- | --- | --- | --- | --- | --- | --- | --- |
| **Quality assessment of retrospective studies** | | | | | | | | |
| **Study** | **Is the case definition adequate?** | **Representativeness of the cases** | **Selection of Controls** | **Definition of Controls** | **Comparability of cases and controls on the basis of the design or analysis** | **Ascertainment of exposure** | **Same method of ascertainment for cases and controls** | **Quality score** |
| Castello et al, 2015 | Yes, e.g., record linkage or based on self-reports  *1017 incident cases of BC diagnosed in the Oncology departments of 23 hospitals members of the Spanish Breast Cancer Research Group* | Consecutive or obviously representative series of cases | Hospital controls    *Each case was matched with a healthy control of similar age (± 5 years), selected from cases’ in-law relatives, friends, neighbors, or work colleagues residing in the same town.* | No history of disease (endpoint)  *Women previously diagnosed with breast cancer and women who were unable to answer the questionnaire due to health, language or educational issues were excluded*. | Study controls for similar age.  *Total calorie intake, smoking habit, age at first delivery, education, history of breast problems, family history of BC and menopausal status.* | Written self-report  *Adjusted for total calorie intake, smoking habit, age at first delivery, education, history of breast problems, family history of BC. Menopausal status, age and hospital. OR and 95% CI for WRFC/AIRC score. Cases and controls completed a structured and self-administered questionnaire. Dietary intake in the last five years was estimated using a 117-item semi-quantitative food frequency questionnaire (FFQ) adapted to and validated in different Spanish adult populations* | Yes | Good  ★★★★★★★ |
|  | ★ | ★ |  | ★ | ★★ | ★ | ★ |  |
| Fainidi et al, 2015 | Yes, e.g., record linkage or based on self-reports  *Patients with a new histologically confirmed diagnosis of BC, regardless of the stage of disease* | Consecutive or obviously representative series of cases | Community controls | No history of disease (endpoint)  *Anthropometric measurements, mammography and a blood sample.* | Study controls for age, region and institution.  *Adjusted for age at first pregnancy, number of full-term pregnancies, energy intake, socio-economic status, age at menarche, hormone therapy and family history of BC. Conditioning for matching factors (age, region and health-care institution).* | At least one questionnaire ascertainment of exposure validated was blind to case/control status.  *A trained interviewer administered a questionnaire to each selected participant to collect information on her health, physical activity and diet. FFQ adapted from the Nurses’ Health Study for the Mexican population and validated in Mexico City* | Yes | Good  ★★★★★★★★ |
|  | ★ | ★ | ★ | ★ | ★★ | ★ | ★ |  |
| Ghosn et al, 2020 | Yes, e.g., record linkage or based on self-reports  *By physical examination and mammography findings.* | Consecutive or obviously representative series of cases | Community controls | No history of disease (endpoint)  *Randomly selected from apparently healthy women* | Study controls for age, sex and BMI.  *Model I: Adjusted for age, residence, marital status, SES, education, family history of B. C, menopausal status, breast feeding, history of disease, supplement use, smoking and HEI score. Model II: Further controlled for BMI.* | At least one questionnaire ascertainment of exposure validated was blind to case/control status.  *Interviewed based. Willett- format semi-quantitative dish-based food frequency questionnaire (FFQ) which was designed and validated specifically for Iranian adults.* | Yes | Good  ★★★★★★★★ |
|  | ★ | ★ | ★ | ★ | ★★ | ★ | ★ |  |
| Khalis et al, 2019 | Yes, e.g., record linkage or based on self-reports  *Newly- diagnosed histologically-confirmed in situ or invasive BC* | Consecutive or obviously representative series of cases | Hospital controls  *Controls were randomly selected woman visiting one of six outpatient primary health centers (three urban and three rural) in the Fez region* | No history of disease (endpoint) | Study controls for age and region  *Age, number of live births, menopausal status and age at menopause combined, history of oral contraceptive. family history of breast cancer, wealth score, age at first full-term pregnancy and energy intake.* | At least one questionnaire ascertainment of exposure validated was blind to case/control status.  *Participants were interviewed at enrolment in the study by four trained and standardized interviewers and data were collected using an in-depth questionnaire. Usual dietary intake of participants during the previous 12 months was assessed using a previously validated Food Frequency Questionnaire.* | Yes | Good  ★★★★★★★ |
|  | ★ | ★ |  | ★ | ★★ | ★ | ★ |  |
| McKenzie, et al 2014 | Yes, e.g., record linkage or based on self-reports  *All women with a primary invasive breast cancer registered on the New Zealand Cancer Registry (NZCR) between 1st April 2005 and 30th April 2006 were eligible for inclusion.* | Consecutive or obviously representative series of cases. | Community controls | No history of disease (endpoint) | Study controls for age.  *Adjusted for covariates: parity, age at menarche, history of maternal breast cancer, oral contraceptive use, HRT use, diabetes, SEP, and age.* | At least one questionnaire ascertainment of exposure validated was blind to case/control status.  *Godin Leisure Time Exercise Questionnaire* | Yes | Good  ★★★★★★★★ |
|  | ★ | ★ | ★ | ★ | ★★ | ★ | ★ |  |
| Romaguera et al, 2017 | Yes, e.g., record linkage or based on self-reports  *Histologically-confirmed newly-diagnosed cance*r. MCC-Spain is a multicentric case–control study with population controls and cases | Consecutive or obviously representative series of cases. | Community controls | No history of disease (endpoint) | Study controls for age and sex  *Adjusted for age, educational level, area, family history of prostate cancer, smoking, and total energy intake. All components were mutually adjusted for each other* | At least one questionnaire ascertainment of exposure validated was blind to case/control status.  *Subjects were provided a semiquantitative Food Frequency Questionnaire (FFQ), which was a modified version from a previously validated instrument in Spain* | Yes  ★ | Good  ★★★★★★★★ |
|  | ★ | ★ | ★ | ★ | ★ ★ | ★ | ★ |  |
| Sanchez-Zamorano et al, 2011 | Yes, e.g., record linkage or based on self-reports  *Patients with a new histologically confirmed diagnosis of BC, regardless of the stage of disease.* | Consecutive or obviously representative series of cases | Community controls | No history of disease (endpoint) | Study controls for age.  *OR values are adjusted by age category, health care system, and region, as well as for socioeconomic status, breastfeeding, BMI, family history of breast cancer, history of diabetes, folate consumption in diet, total daily calories consumption, height, and WHR*. | At least one questionnaire ascertainment of exposure validated was blind to case/control status.  *Food Frequency Questionnaire (FFQ) adapted from Willett to the Mexican population and validated in Mexico City* | Yes | Good  ★★★★★★★★ |
|  | ★ | ★ | ★ | ★ | ★★ | ★ | ★ |  |
| ACR: Alberta Cancer Registry AICR: American Institute for Cancer Research; ARIC: Atherosclerosis Risk In Communities; ATP: Alberta’s Tomorrow Project; BC: Breast cancer; BCIS: in situ breast cancer; BMI: body mass index; BWHS: The Black Women’s Health Study; C-DHQ: Canadian Diet History Questionnaire; CCR: Canadian Cancer Registry; CHC: community health centers; CI: 95% confidence interval; CT: clinical trial; DQX: Dietary questionnaire; EBBA-Life: Energy Balance and Breast Cancer Aspects throughout life; EPIC: European Prospective Investigation into Cancer and Nutrition; FFQ: Food Frequency Questionnaire; HEI: Healthy Eating Index; HR: Hazard ratio; HRT: hormone replacement therapy; HT: hormonal replacement therapy; IWHS: Iowa Women's Health Study; N: number; NBSS: Canadian National Breast Screening Study; NCI: National Cancer Institute's; NHS: National Health Service; NIH-AARP: American Association of Retired Persons; NIH: National Institutes of Health; NOWAC: Norwegian Women and Cancer; NSAID: nonsteroidal anti-inflammatory drug; NZCR: New Zealand Cancer Registry; OR: odds ratio; OS: observational study; PLCO: Prostate, Lung, Colorectal, and Ovarian Cancer Screening Trial; PMH: postmenopausal hormone; SCCS: Southern Community Cohort Study; SEER: Surveillance, Epidemiology and End Results; SES: socio-economic status; SMC: Swedish Mammography Cohort; SUN: Seguimiento Universidad de Navarra; TEI: total energy intake; UK: United Kingdom; VITAL: The Vitamins and Lifestyle study; WCRF: World Cancer Research Fund; WHI-OS: Women’s Health Initiative Observational Study; WHR: Waist-to-hip ratio.  ^a^For prospective studies, the Selection domain includes: a) Representativeness of the exposed cohort, b) Selection of the non-exposed cohort, c) Ascertainment of exposure and d) Demonstration that outcome of interest was not present at start of study; Comparability domain: a) Comparability of cohorts on the basis of the design or analysis controlled for confounders; Outcome domain: a) Assessment of outcome, b) Was follow-up long enough for outcomes to occur and c) Adequacy of follow-up of cohorts  ^b^For retrospective studies, the Selection domain includes a) ¿Is the case definition adequate, b) Representativeness of the cases, and c) Selection of Controls; Comparability domain: a) Comparability of cases and controls on the basis of the design or analysis; Outcome domain: a) Ascertainment of exposure and b) Same method of ascertainment for cases and controls. | | | | | | | | |

| **Supplemental Table 3.** Summary of findings of the comparison of the highest adherence to healthy lifestyle index compared to the lowest adherence to healthy lifestyle index for breast cancer (GRADE). | | | | |
| --- | --- | --- | --- | --- |
| **Outcomes** | **Relative effect (95% CI)** | **№ of participants (studies)** | **Certainty of the evidence (GRADE)** | **Comments** |
|  |  |  |  |  |
| Overall hazard ratio of breast cancer over healthy lifestyle index in prospective studies assessed with: Hazard ratio follow-up: range 5 years to 23 years | **HR 0.80** (0.77 to 0.83) | 37,675 (14 observational studies) | ⨁⨁⨁◯ Moderate | Highest adherence to a healthy lifestyle index likely reduces overall hazard ratio of breast cancer over the lowest category of adherence to a healthy lifestyle index in prospective studies. |
| Overall odds ratio of breast cancer over healthy lifestyle index in retrospective studies assessed with: Odds ratio | **OR 0.74** (0.63 to 0.86) | 3,596 cases  (4 observational studies) | ⨁⨁◯◯ Low^a^ | Highest adherence to a healthy lifestyle index reduces overall odds ratio of breast cancer over the lowest category of adherence to a healthy lifestyle index in retrospective studies. |
|  |  |  |  |  |
| Pre-menopausal hazard ratio of breast cancer over healthy lifestyle index in prospective studies assessed with: hazard ratio follow-up: range 5 years to 23 years | **HR 0.96** (0.88 to 1.03) | 6,319 cases (6 observational studies) | ⨁◯◯◯ Very low^b^ | The evidence is very uncertain about the effect of highest adherence to a healthy lifestyle index on pre-menopausal hazard ratio of breast cancer over the lowest category of adherence to a healthy lifestyle index in prospective studies. |
| Post-menopausal hazard ratio of breast cancer over healthy lifestyle index in prospective studies assessed with: Hazard ratio | **HR 0.78** (0.76 to 0.81) | 32,338 cases (12 observational studies) | ⨁⨁⨁◯ Moderate | Highest adherence to a healthy lifestyle index likely results in a reduction in post-menopausal hazard ratio of breast cancer over the lowest category of adherence to a healthy lifestyle index in prospective studies. |
| Pre-menopausal odds ratio of breast cancer over healthy lifestyle index in retrospective studies assessed with: Odds ratio | **OR 0.74** (0.59 to 0.92) | 2,289 cases  (6 observational studies) | ⨁⨁⨁◯ Moderate | Highest adherence to a healthy lifestyle index probably results in a reduction in pre-menopausal odds ratio of breast cancer over the lowest category of adherence to a healthy lifestyle index in retrospective studies. |
|  |  |  |  |  |
| Post-menopausal odds ratio of breast cancer over healthy lifestyle index in retrospective studies assessed with: Odds ratio | **OR 0.57** (0.47 to 0.68) | 3,174 cases  (6 observational studies) | ⨁⨁⨁⨁ High | Highest adherence to a healthy lifestyle index results in large reduction in post-menopausal odds ratio of breast cancer over the lowest category of adherence to a healthy lifestyle index in retrospective studies. |
|  |  |  |  |  |
| Overall hazard/odds ratio of breast cancer molecular subtype ER+/PR+ over healthy lifestyle index | **HR/OR 0.68** (0.63 to 0.73) | 5,905 cases (6 observational studies) | ⨁⨁⨁◯ Moderate | Highest adherence to a healthy lifestyle index likely reduces overall hazard/odds ratio of breast cancer molecular subtype ER+/PR+ over the lowest category of adherence to a healthy lifestyle index. |
|  |  |  |  |  |
| Overall hazard/odds ratio of breast cancer molecular subtype ER+/PR- over healthy lifestyle index | **HR/OR 0.78** (0.67 to 0.90) | 1,490 cases (3 observational studies) | ⨁⨁⨁◯ Moderate | Highest adherence to a healthy lifestyle index probably results in a reduction in overall hazard/odds ratio of breast cancer molecular subtype ER+/PR- over the lowest category of adherence to a healthy lifestyle index. |
|  |  |  |  |  |
| Overall hazard/odds ratio of breast cancer molecular subtype ER-/PR- over healthy lifestyle index | **HR/OR 0.77** (0.64 to 0.92) | 1,619 cases (5 observational studies) | ⨁⨁⨁◯ Moderate | Highest adherence to a healthy lifestyle index probably results in a reduction in overall hazard/odds ratio of breast cancer molecular subtype ER-/PR- over the lowest category of adherence to a healthy lifestyle index |
|  |  |  |  |  |
| N: number; HR: hazard ratio; OR: odds ratio; 95% CI: confidence interval; ER: estrogen receptor; PR: progesterone receptor; GRADE: Grading of Recommendations, Assessment, Development and Evaluations.  a. Downgraded heterogeneity I^2^= 90.8%  b. Downgraded because slight or not association was observed | | | | |


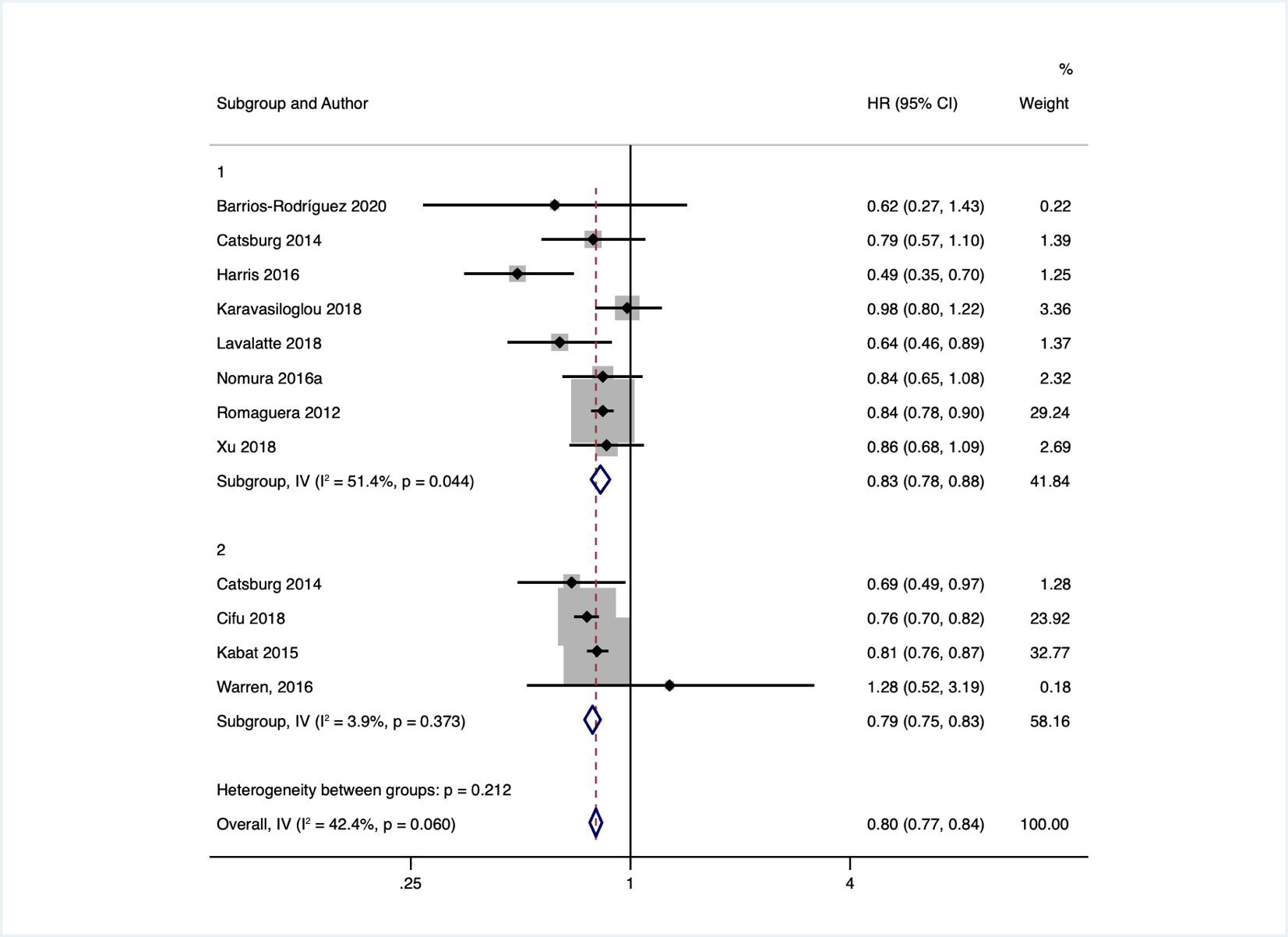
**Sensitivity analysis**

ACS

WCRF/AICR

**Supplemental Figure 1**. Sensitivity analyzes of prospective studies that included a healthy lifestyle index based on the guidelines for cancer prevention such as the WCRF/AICR or ACS.

**
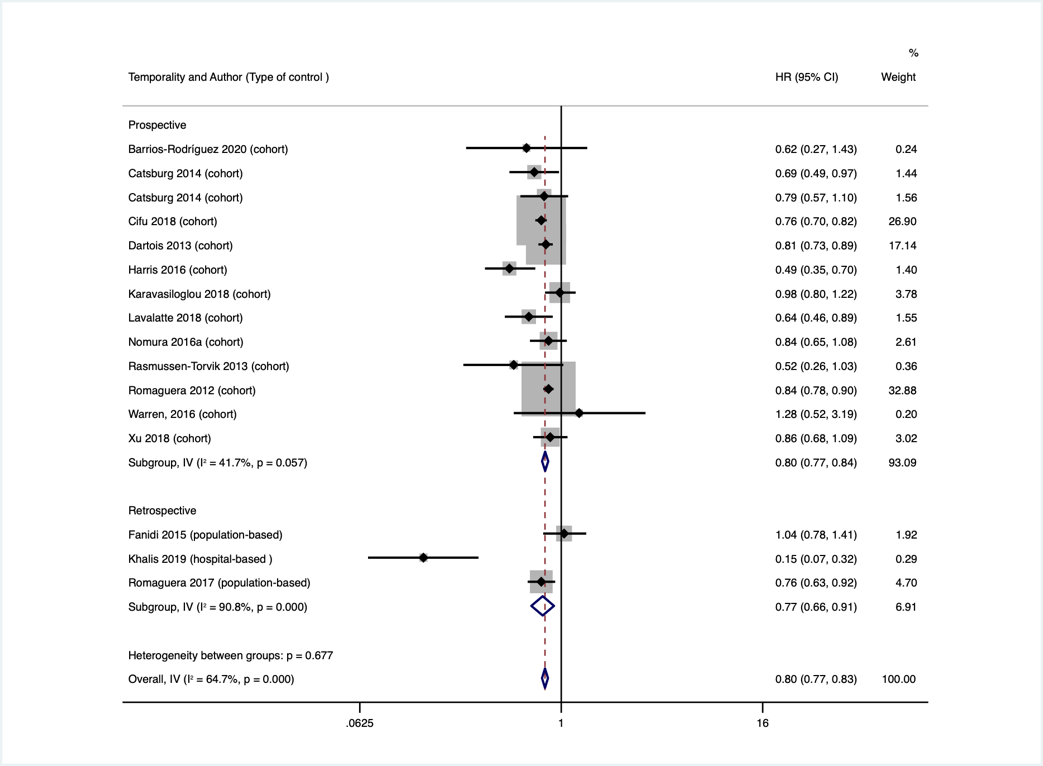

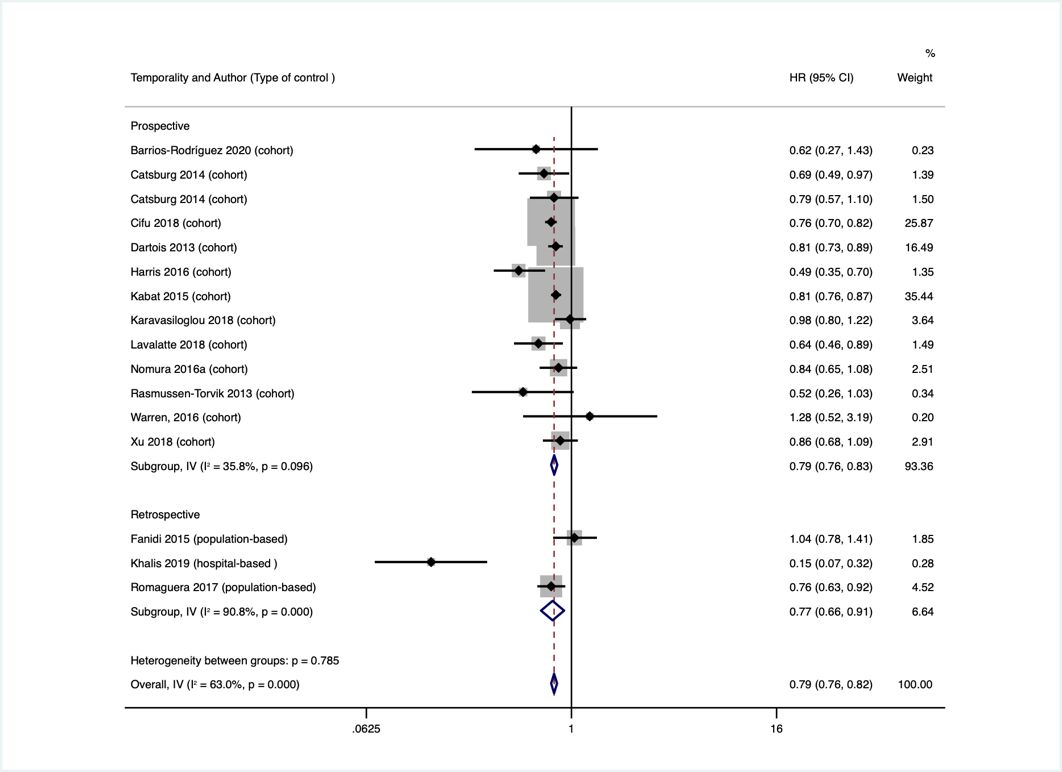
**

b)


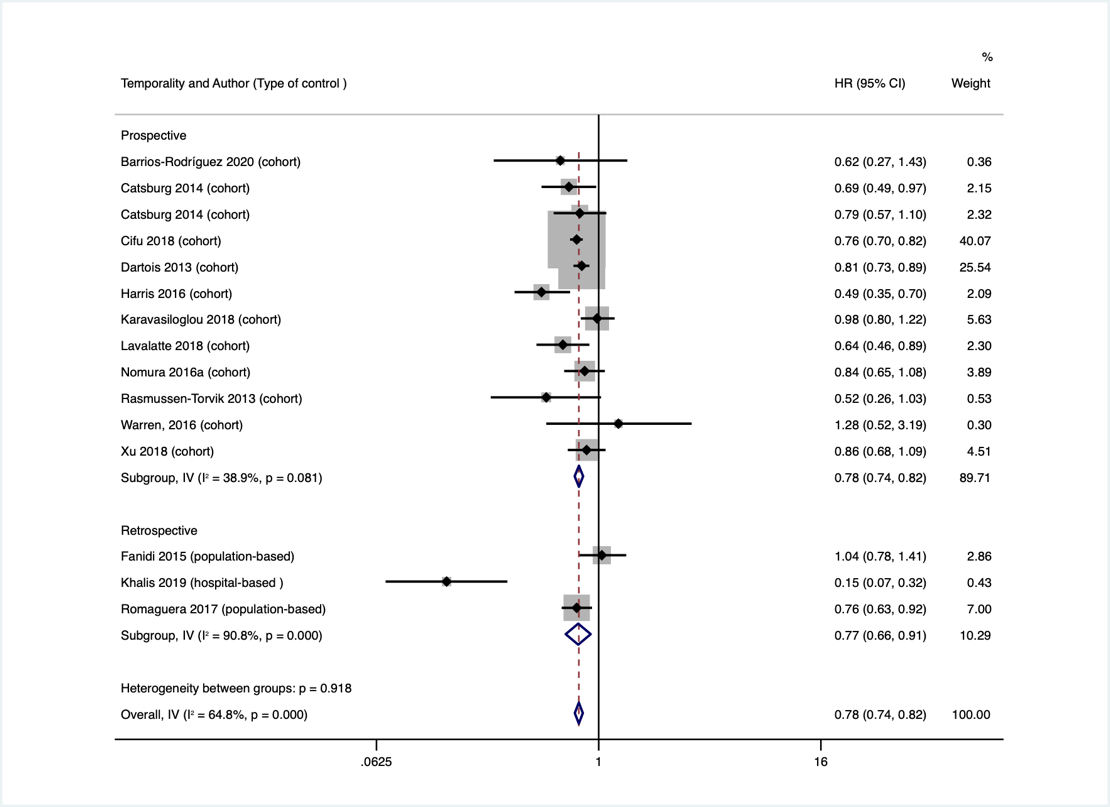


**Supplemental Figure 2**. Sensitivity analyzes of prospective studies excluding those studies that represented a greater weight in the model of random effects. a) Sensitivity analysis excluding the study by Romaguera, 2012. b) Sensitivity analysis excluding the study by Kabat, 2015. c) Sensitivity analysis excluding both studies by Romaguera, 2012 and Kabat, 2015.

**Publication bias**

**
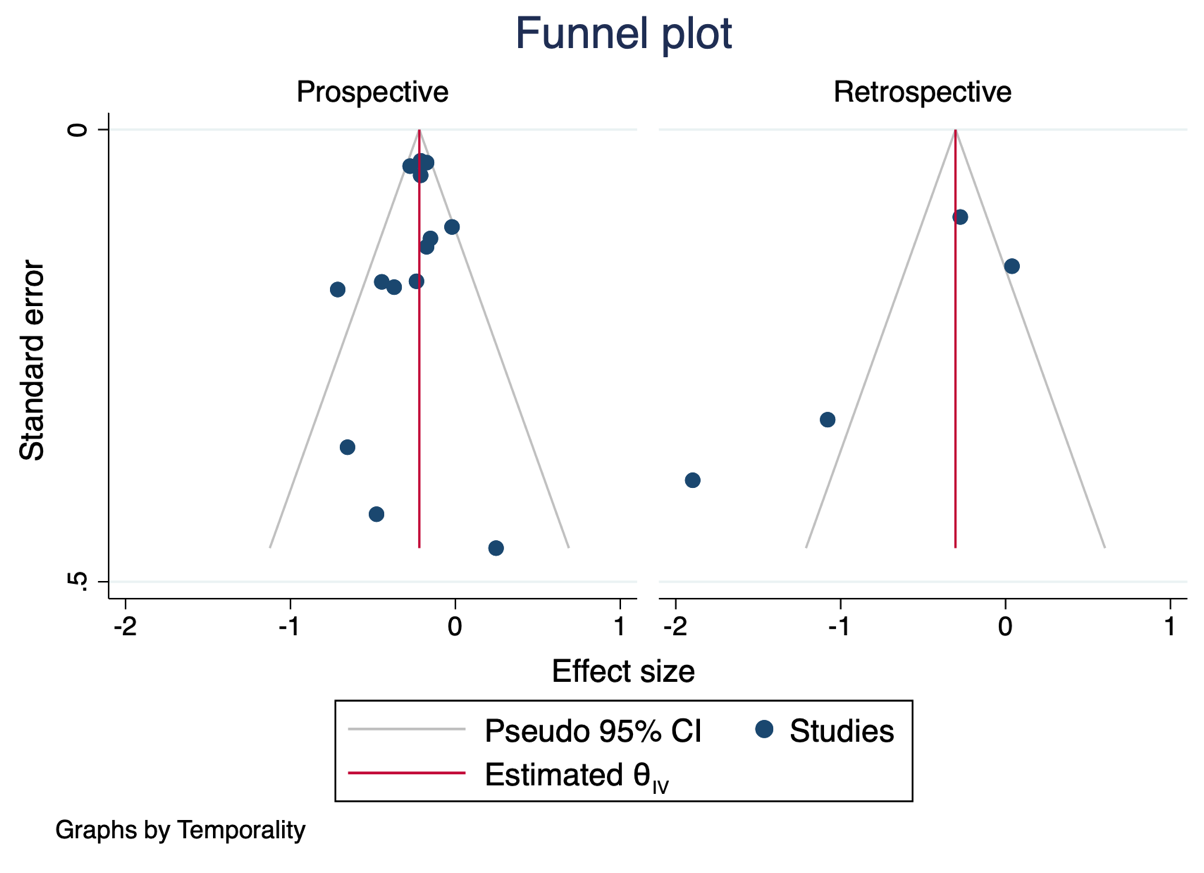
**

**Supplemental Figure 3.** Publication bias analysis of the studies included in the meta-analysis stratified by study design: prospective and retrospective studies.


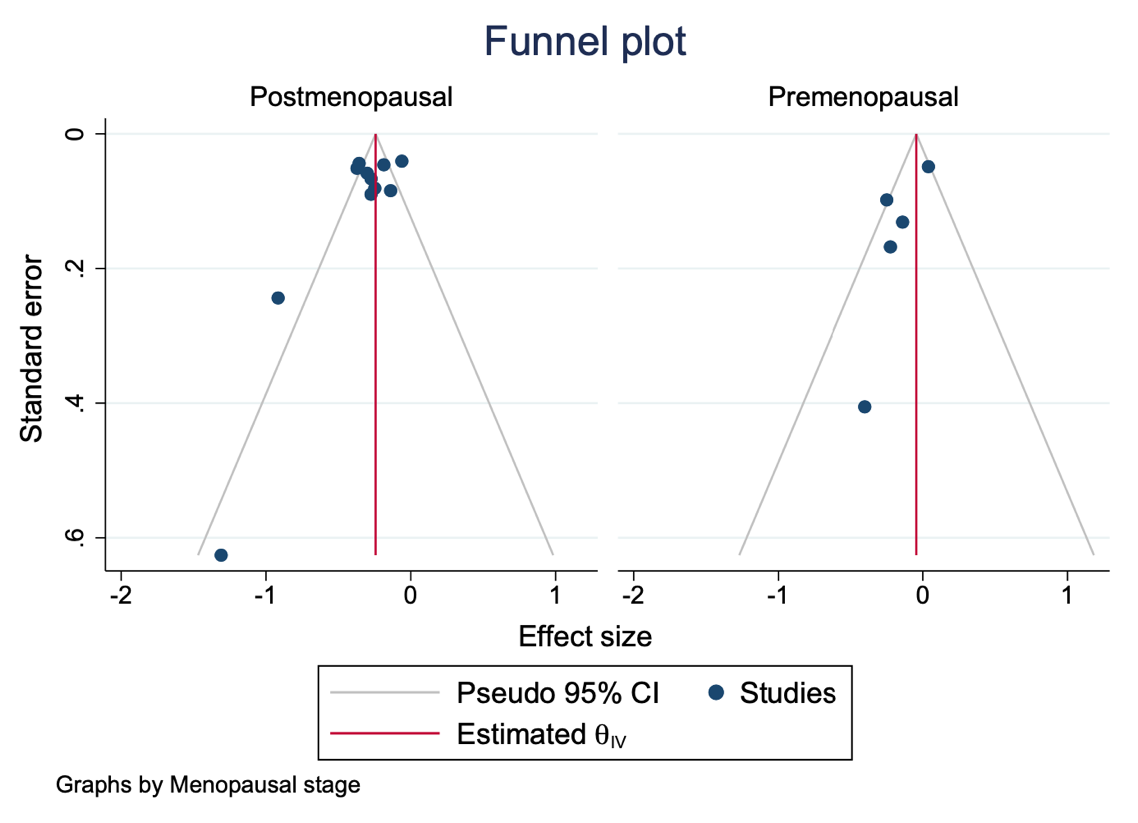


**Supplemental Figure 4.** Publication bias analysis of prospective studies included in the meta-analysis stratified by menopausal status: premenopausal and postmenopausal.

**
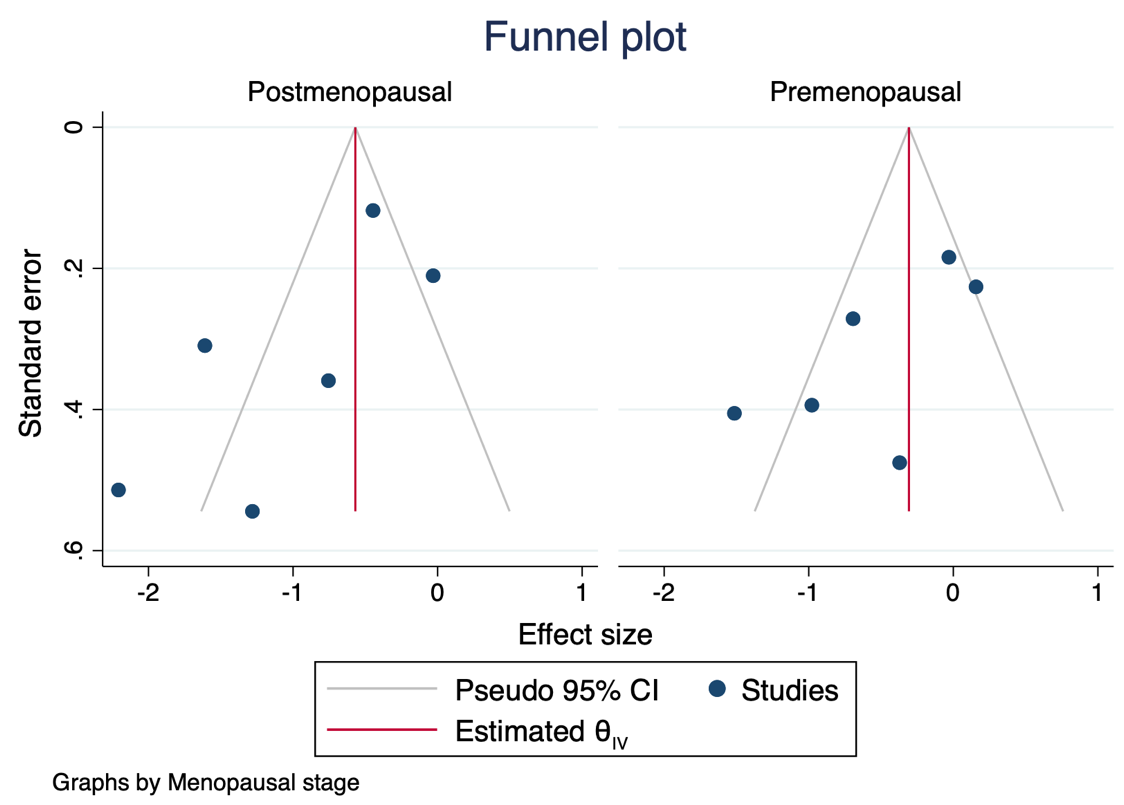
**

**Supplemental Figure 5.** Publication bias analysis of retrospective studies included in the meta-analysis stratified by menopausal status: premenopausal and postmenopausal.


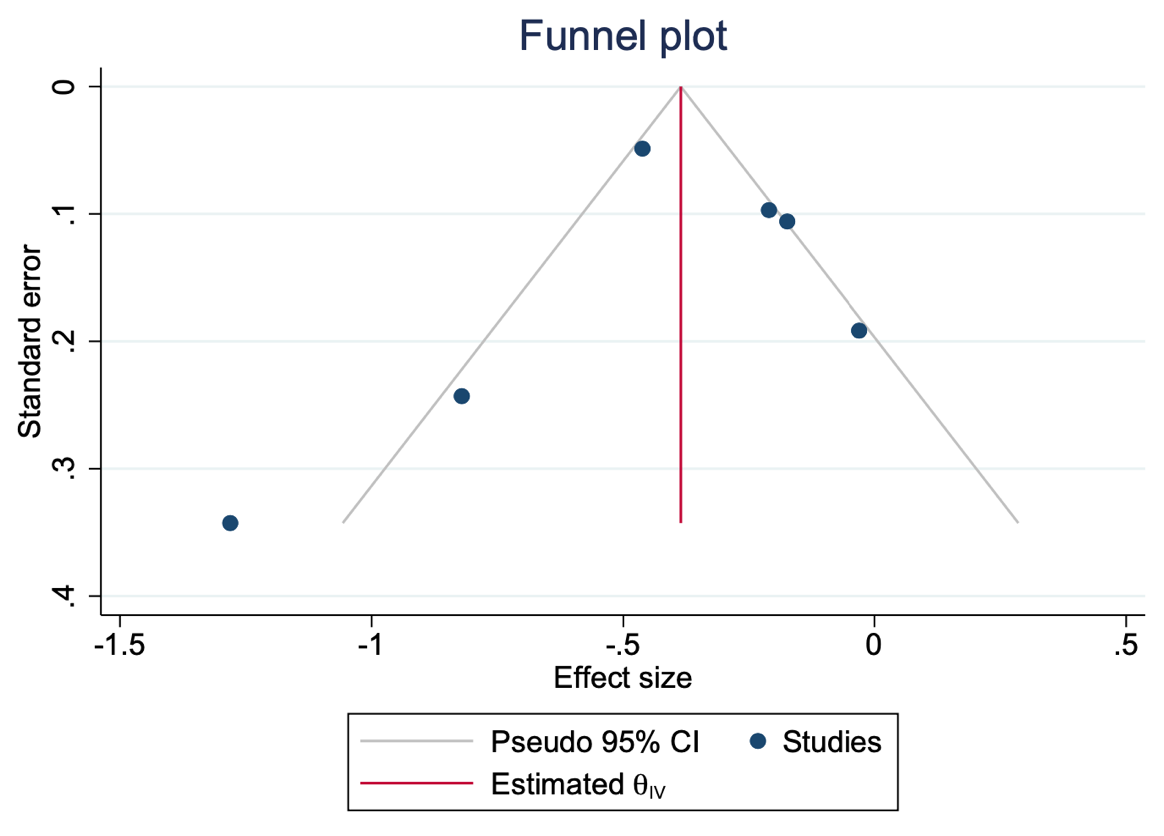


**Supplemental Figure 6.** Publication bias analysis of prospective and retrospective studies included in the meta-analysis stratified breast cancer molecular subtype: ER+/PR+.

**Supplemental Figure 7.** Publication bias analysis of prospective and retrospective studies included in the meta-analysis stratified breast cancer molecular subtype: ER+/PR-


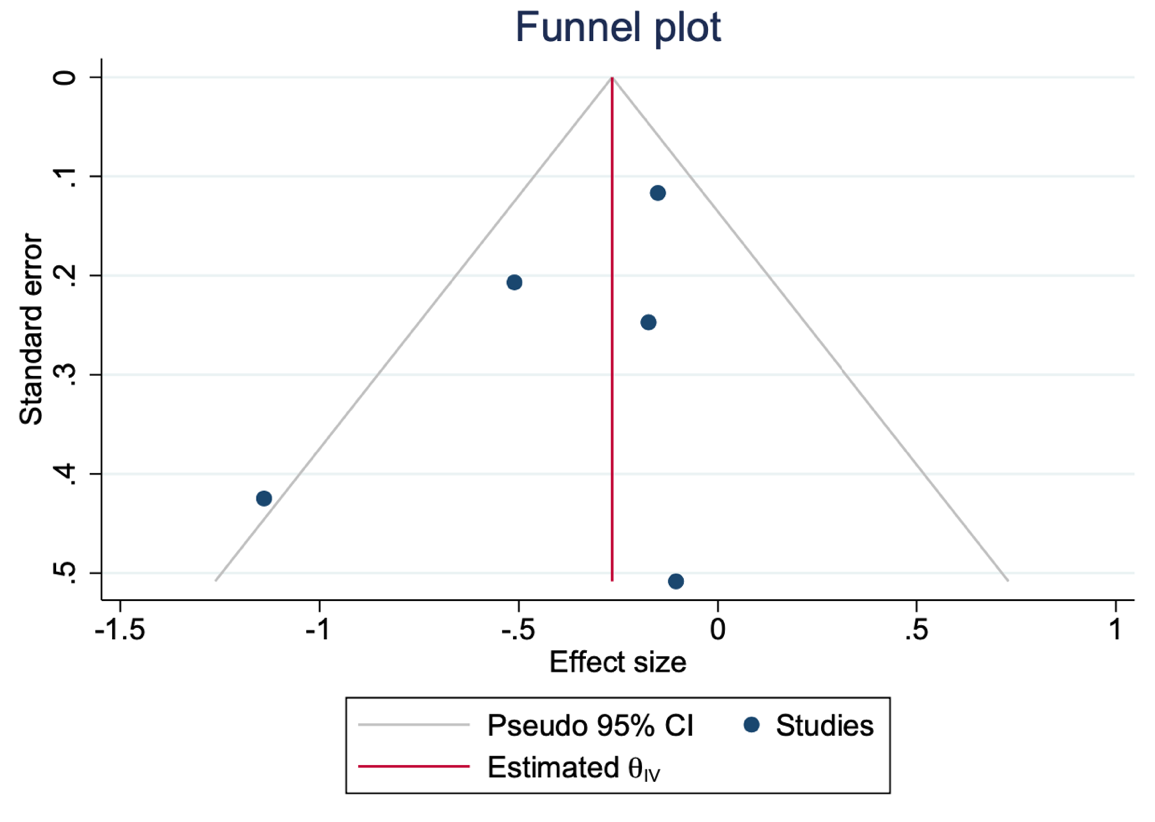


**Supplemental Figure 8.** Publication bias analysis of prospective and retrospective studies included in the meta-analysis stratified breast cancer molecular subtype: ER-/PR-
